# Advanced Ann Arbor stage and age over 60 years as prognostic predictors in patients with primary cervical lymphoma: a retrospective cohort study and systematic review

**DOI:** 10.1186/s12885-023-10548-4

**Published:** 2023-01-27

**Authors:** Lingyun Gao, Xiaoran Chen, Jing Zhao, Anli Xu, Meijuan Liu, Hongna Yu, Shujun Kong, Sijian Li

**Affiliations:** 1 Department of Ultrasound, The Affiliated Yantai Yuhuangding Hospital of Qingdao University, Yantai, Shandong People’s Republic of China; 2 Department of Hematology, The Affiliated Yantai Yuhuangding Hospital of Qingdao University, Yantai, Shandong People’s Republic of China; 3 Department of Obstetrics and Gynecology, The Affiliated Yantai Yuhuangding Hospital of Qingdao University, Yantai, Shandong People’s Republic of China; 4 Department of Obstetrics and Gynecology, Peking Union Medical College Hospital, Chinese Academy of Medical Sciences, Peking Union Medical College, Beijing, People’s Republic of China

**Keywords:** Extranodal lymphoma, Primary cervical lymphoma, Diffuse large B-cell lymphoma, Prognosis, Risk factors

## Abstract

**Objective:**

To evaluate the overall survival (OS), disease-specific survival (DSS), and recurrence-free survival (RFS) for primary cervical lymphoma (PCL), an extremely rare disease without treatment consensus.

**Methods:**

We conducted a retrospective study included 177 patients, including 169 cases identified from literature review. The Kaplan-Meier methods and Cox regression were used to determine the OS, DSS, RFS, and relevant risk factors.

**Results:**

The 5-year OS and 5-year DSS rates were 85.8 and 87.2%, respectively, while the 5-year RFS rate was 85.5%. Diffuse large B-cell lymphoma (DLBCL) was the predominant subtype that comprised 63.8% (113 cases) in this cohort. Multivariate analysis in the DLBCL subgroup revealed that age ≥ 60 years (Odds ratio [OR]: 26.324, 95% Confidence Interval [CI]: 5.090–136.144, *P* < 0.001) or stage IIIE-IVE (advanced stage) (OR: 4.219, 95%CI: 1.314–13.551, *P* = 0.016) were the risk factors for OS, while patients with age ≥ 60 years (OR:23.015, 95%CI: 3.857–137.324, *P* = 0.001), and stage IIIE-IVE (OR: 4.056, 95% CI: 1.137–14.469, *P* = 0.031) suffered a poor DSS. Chemotherapy and/or radiotherapy improved the OS (*P* = 0.008), DSS (*P* = 0.049), and RFS (*P* = 0.003). However, cancer-directed surgery did not improve the OS, DSS, and RFS. The risk factor was unavailable in other subtypes of PCL due to limited cases.

**Conclusion:**

The survival outcomes in patients with PCL at early stage were satisfactory, while the advanced disease stage and age ≥ 60 years were the two major factors predicting poor prognosis in DLBCL subtype.

**Supplementary Information:**

The online version contains supplementary material available at 10.1186/s12885-023-10548-4.

## Introduction

Primary lymphoma that originates from a body organ or tissue other than lymph nodes or spleen is classified as the primary extranodal lymphoma (PEL), accounting for 25–40% of all the primary lymphoma cases [1]. Although PEL mostly involves the gastrointestinal (GI) tract, central nervous system (CNS), bone, while breast, skin, and testis have also been reported to be the primary sites of lymphomagenesis [2]. Among all the PEL cases, involvement of the female genital tract is clinically rare (~ 1%), with only approximately 300 reported cases of primary cervical lymphoma (PCL) in English literatures [3–5]. Patients with PCL usually present unspecified manifestations, such as abnormal uterine bleeding, that may be misdiagnosed for other common cervical diseases [6]. Due to its rarity, there is no consensus management of PCL. Notably, effective treatment options are mainly derived from case reports and narrative reviews [6–9]. Moreover, data on the survival outcomes and prognosis predictive factors remain insufficient.

Several research has investigated the treatment options and survival outcomes in patients with PCL. Evidence indicates the application of chemotherapy with or without radiotherapy in accordance with the guidelines for extranodal lymphoma as the preferred management [3, 6, 9, 10], avoiding the surgical interventions [3, 5]. Kosari et al. summarized the clinicopathological characteristics of the female genital tract lymphoma in 186 patients, where only 17 cases involved the uterine cervix, and 37% of total cases were secondary lymphoma [4]. Both Harris et al. and Ahmad et al. reported a 5-year survival rate of 73–86% in small case series of PCL [11, 12]. Additionally, Mandato et al. in 2014 and Hilal et al. in 2016 successively revealed a comparable overall survival (OS) rate of about 80% in two large cohorts, including more than 100 cases of PCL [3, 6]. However, none had evaluated the confounding risk factors for OS and lymphoma recurrence. Recently, it has been revealed that a 5-year OS rate and cancer-specific survival rate of over 70% in 697 patients and proposed potential prognostic factors [5]. Nonetheless, only 21.4% of patients in this cohort had PCL.

The objective of this study was to investigate the clinical characteristics and survival outcomes in patients with PCL, while also evaluating the risk factors of the OS, disease-specific survival (DSS), and recurrence-free survival (RFS).

## Materials and methods

The Ethics Committee of the Yantai Yuhuangding Hospital approved this study. First, we retrospectively reviewed 8 cases of PCL treated in Yantai Yuhuangding Hospital between 2010 and 2022. Then a literature review of studies on PCL published between 1980 and 2022 was conducted to select eligible full-length reports in the English language. The keywords used for searching in the PubMed, Embase, and Scopus were as follows: “primary cervical lymphoma”; “primary lymphoma of the uterine cervix”; “primary cervical Hodgkin’s lymphoma”; “primary cervical non-Hodgkin’s lymphoma (NHL)”; “primary lymphoma of female genital tract”; “extranodal lymphoma”. Relevant references cited within these articles were also reviewed. The exclusion criteria included studies on patients with PEL of other sites, cases reported by letters or personal opinions, non-English literature, secondary cervical lymphoma, imaging studies on PCL, and reports with insufficient data on clinical characteristics and/or follow-up results. All the eligible studies were enrolled for final analysis. We included 169 cases of PCL reported in 86 studies following the screening according to the PRISMA (For details, see Supplementary Fig. S1). The eight cases treated in our hospital were also incorporated into the analysis. Finally, we established a database of 177 patients, including their demographic and clinical characteristics, treatment strategies, and survival outcomes (Table S1).

We conducted subgroup survival analysis of diffuse large B-cell lymphoma (DLBCL) because DLBCL was an aggressive lymphoma that predominant in this population. Other PCL pathologic subtypes were briefly analysis due to the limited cases. Clinical characteristics were analyzed to identify independent predictors of OS, DSS, and RFS, including age (< 60, ≥60 years, the cut-point was selected reference to the NCCN-IPI [13]), Ann Arbor stage (stage IE-IIE or IIIE-IVE), cancer-directed surgery (yes or no), and chemo/radiotherapy (no treatment, single treatment, or combination therapy). Cancer-directed surgery (CDS) referred to surgeries that were aimed to cure the disease rather than collecting biopsy samples, such as radical trachelectomy, hysterectomy with/without bilateral salpingo-oophorectomy (H/BSO), and extended surgical resections (radical H/BSO plus retroperitoneal lymph nodes resection). The 2016 revision of the WHO classification of lymphoid neoplasms did not directly define the PCL. However, according to the classification of lymphoma based on anatomical sites, PCL can be classified as a subtype of PEL. Krol et al. [14] proposed to use a liberal definition of primary extranodal NHL that includes all patients who present with NHL that apparently originated at an extranodal site, even in the presence of disseminated disease, as long as the extranodal component is clinically dominant. As most of the women presented lymphoma of cervix would visit gynecologic physicians, we think that retain the concept of PCL, this special situation, including localized and disseminated disease, and identifying the survival outcomes and risk factors are important and make sense. Therefore, we used the liberal definition of PEL described as Krol et al. [14]. The disease stage was classified based on the Ann Arbor staging system for extranodal lymphoma [7]. RFS was defined as the date from initial treatment intervention to confirmed tumor recurrence. OS was defined as the time from the date of initial diagnosis to death associated with any cause or the last follow-up. DSS was defined as the time from the date of the initial diagnosis to death related to PCL or the final follow-up.

### Statistical analysis

Means ± standard deviation (SD, range) and medians and interquartile ranges (IQRs) were used to describe normally distributed continuous variables according to their distributions. Counts (percentages) were used to express discrete variables. Categorical variables were compared by the chi-squared (χ^2^) test or Fisher’s exact test. Survival analyses were performed using the Kaplan-Meier (log-rank test) analysis. Univariate analyses for OS, DSS, and RFS were performed to screen variables for further evaluation in multivariate models. Factors with *P*-value < 0.1 were included further in the multivariate analysis using the Cox regression model to identify potential independent prognostic predictors. A two-tailed *P*-value < 0.05 was considered statistically significant. We used SPSS (version 21.0; SPSS Inc., Chicago, IL, USA) or GraphPad Prism (version 8.0; GraphPad Software Inc., San Diego, CA, USA) software, wherever appropriate, to conduct statistical analyses.

## Results

### Eight cases of PCL treated in Yuhuangding hospital

#### Demographic and clinical characteristics

**Fig. 1:**
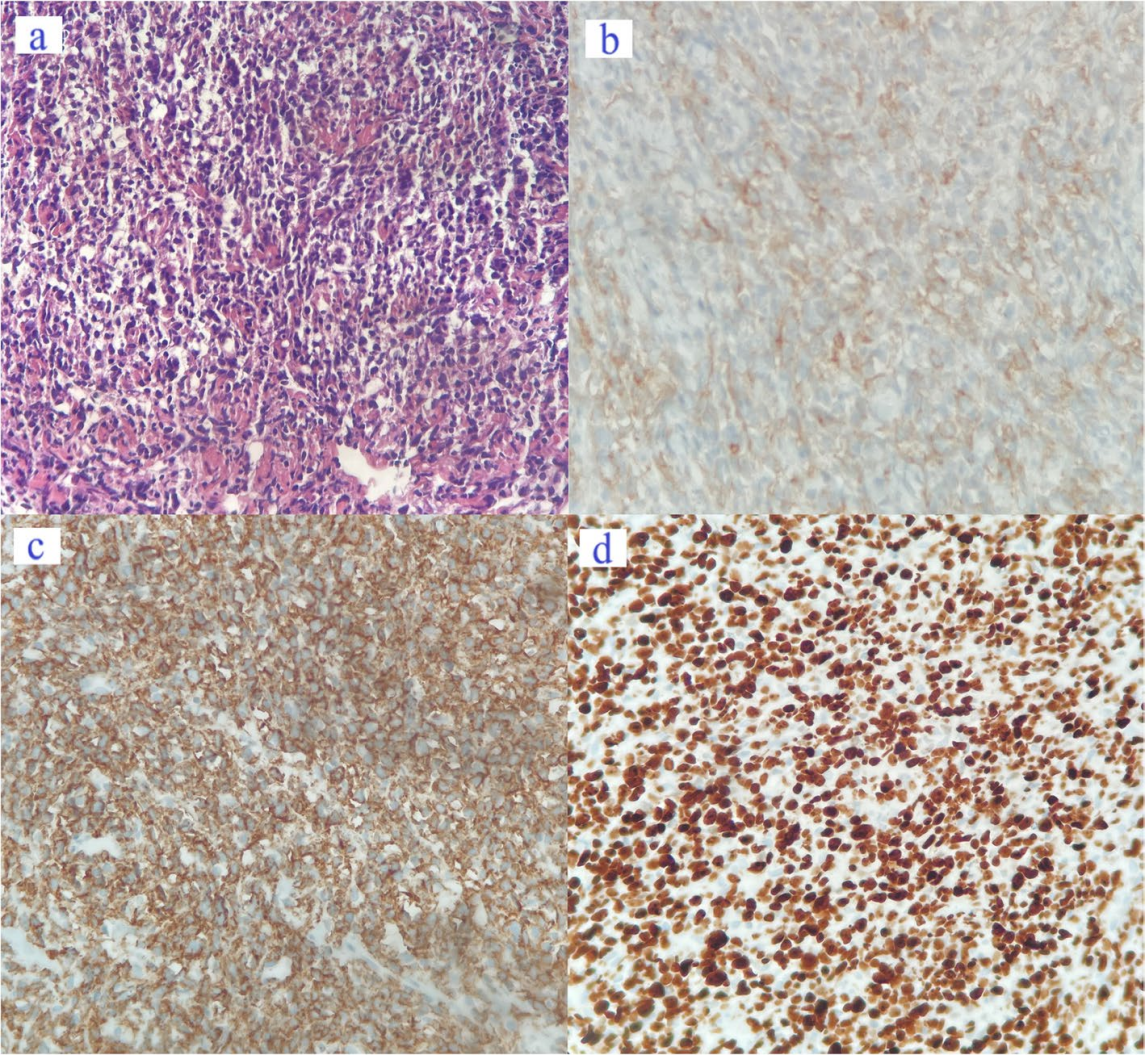
Pathological images of patients with cervical diffuse large B-cell lymphoma (DLBCL) (400X magnification in four figures, case 6). **a** H&E staining demonstrated diffused infiltration of numerous lymphoid cells, with large cell size, bright cytoplasm, and irregular nuclei, supporting the diagnosis of DLBCL (germinal center type). **b** Immunohistochemical (IHC) staining indicated the CD10-positive status. **c** Diffused CD20 positivity was revealed by IHC. **d** The Ki67 index was approximately 80%

We retrospectively reviewed 8 cases of PCL treated between 2010 and 2022. The median age of these patients was 60.0 years (range: 31–72). At the time of diagnosis, six patients had Ann Arbor stage IVE disease and other two patients had Ann Arbor stage IE disease. All of them were classified as non-Hodgkin’s lymphoma (NHL), including 7 cases of DLBCL and one patient was diagnosed with Burkitt lymphoma. The elevated lactate dehydrogenase (LDH) level was noted in 75% of patients, with an average of 537.5 U/L. A representative pathology was presented (Fig. 1, case 6).

#### Surgical intervention, chemotherapy, and radiotherapy

After a suspected diagnosis of cervical malignancy, systemic evaluations, including cervical biopsy, and laboratory and imaging examinations, were conducted. Only one patient (case 3) underwent CDS for the biopsy that revealed uncertain malignancy. The R-CHOP (rituximab, cyclophosphamide, hydroxydaunorubicin, vincristine, and prednisone) therapy was the most commonly used treatment that was applied in five patients. While the R-EPOCH (rituximab, etoposide, prednisone, vincristine, cyclophosphamide, and hydroxydaunorubicin) regimen was administered only in case 3 and the R-hyperCVAD (cyclophosphamide, vincristine, adriamycin, and dexamethasone) in case 5. Moreover, three patients also received intrathecal injections of chemotherapeutic drugs. Furthermore, chemotherapy patients received chemotherapy no less than 5 cycles, and external beam radiation therapy was given to one patient (case 2). However, there was one patient (case 4) who refused any further treatment after diagnosis.

#### Results of follow-up

Five patients achieved complete remission (CR) after initial remission. Among them, one patient (case 5) experienced cancer recurrence in the thoracic and lumbar vertebra at 5 months. After administrations of R-hyperCVAD with intrathecal injection of Ara-C and dexamethasone, for four cycles, the patient obtained partial remission with a stable disease condition. The patient who refused treatment succumbed to rapidly progressive disease at 1.5 months. Partial remission (PR) was also achieved in the other two patients at 5 and 21 months, respectively. However, the progression was uncontrolled at the later stage, eventually leading to death, even after the salvage chemotherapy in one patient (Table 1).

**Table 1:** Patients with primary cervical lymphoma treated in Yuhuangding Hospital

Patients	Age (y)	Manifestations	LDH (U/ml)	Treatment	Pathology	Stage	R	Treatment at R	Result of follow-up
1	66	Hematuria	543	R-CHOP*6	DLBCL	IVE	Not CR	Partial remission and stable disease for 5 months then disease progress and die	DOD at 8 m
2	72	Back pain	356	R-CHOP*5, EBRT	DLBCL	IE	N		NED at 97 m
3	56	Abdominal pain	289	TAH + BSO + pelvic and para-aortic lymphadenectomy; R-EPOCH*9, with intrathecal injection of Ara-C + DXM for 3 cycles	DLBCL	IVE	N		NED at 12 m
4	62	Pelvic mass	861	Refusing treatment	DLBCL	IVE	Not CR	Rapidly progress and die	DOD at 1.5 m
5	31	Lower limb pain	956	R-hyperCVAD*3, R-MA*2, CVP-R*1	Burkitt lymphoma	IVE	Y	R-hyperCVAD*4 with intrathecal injection of Ara-C + DXM, partial remission	AWD at 6 m
6	58	Back pain	280	R-CHOP*8	DLBCL	IVE	N		NED at 27 m
7	71	AUB	586	R-CHOP*6 (4 with Ara-C + DXM intrathecal injection)	DLBCL	IVE	Not CR	Partial remission and stable disease for 21 months, then disease progress, administered with R-CHOP*8, Oxaliplatin + gemcitabine for 3 cycles	DOD at 38 m
8	58	AUB	429	R-CHOP*8 (Ara-C + MTX intrathecal injection *4)	DLBCL	IE	N		NED at 88 m

Abbreviations: *AUB* abnormal uterine bleeding, *LDH* lactate dehydrogenase, *DLBCL* diffuse large B-cell lymphoma, *R* recurrence, *NED* no evidence of disease, *CR* complete remission, *AWD* alive with disease, *DOD* die of the disease

*R-CHOP*: rituximab plus cyclophosphamide, doxorubicin, vincristine, and prednisone, *R-hyperCVAD* rituximab plus cyclophosphamide, doxorubicin, vincristine, and dexamethasone, *DXM* dexamethasone, *MTX* methotrexate, *TAH + BSO* total abdominal hysterectomy plus bilateral salpingo-oophorectomy, *R-EPOCH* rituximab plus etoposide, adriamycin, vincristine, cyclophosphamide, and prednisone

At the final follow-up, four patients achieved no evidence of disease, 3 patients died of the disease, and one patient is still alive with the disease with a median time of 19.5 months (range: 1.5–97 months).

### Literature review cases

#### Overall population

The median age of patients was 49.0 years (range: 20–85), with a median follow-up interval of 2.9 years (mean: 4.2 years, range: 0.08–20.50). Abnormal uterine bleeding was the most common manifestation among them (98/177, 55.4%), and other symptoms included non-specific abdominal pain, abnormal vaginal discharge, etc. Only 12 patients (6.8%) were reported with “B symptoms” and 57.1% of the 28 patients reported elevated LDH level. Tumor sizes were available for 106 patients, with a mean maximal tumor size of 6.9 ± 3.1 cm (range: 0.5–18.0).

More than half (63.8%) of the cases had been classified as Ann Arbor stage IE disease, followed by stage IIE (23.2%), stage IVE (10.7%), and stage IIIE (2.3%), in the descending order. DLBCL was the most predominant pathological subtypes that comprised 63.8% (113 cases) of all the patients in this cohort. Other subtypes such as follicular lymphoma, mucosa-associated lymphoid tissue (MALT) lymphoma, Natural Killer/T-cell lymphoma, and Burkitt lymphoma were much less common. Only 3 (1.7%) could be classified as Hodgkin’s lymphoma (Table 2).

**Table 2:** Clinical characteristics of patients with primary cervical lymphoma (*N* = 177)

Clinical characteristics	Number (Percentile)
Age (Mean/Median, y)	48.9 ± 15.8/49.0 (20–85)
Time of follow-up (Mean/Median, y)	4.2/2.9 (0.08–20.50)
Maximum Tumor size (*N* = 106)	6.9 ± 3.1 (0.5–18.0)
Elevated LDH (*N* = 28)	16 (57.1%)
B symptoms	12 (6.8%)
Ann Arbor Stage	
IE	113 (63.8%)
IIE	41 (23.2%)
IIIE	4 (2.3%)
IVE	19 (10.7%)
Pathological subtypes	
DLBCL	113 (63.8%)
Follicular lymphoma	14 (7.9%)
NK/T-cell lymphoma	3 (1.7%)
Burkitt lymphoma	1 (0.6%)
MALT lymphoma	4 (2.3%)
B-cell NHL, unspecified	14 (7.9%)
Other NHL, unspecified	25 (14.1%)
Hodgkin lymphoma, unspecified	3 (1.7%)
Cancer-directed surgery	59 (33.3%)
Chemotherapy/Radiotherapy details	
No chemotherapy or radiotherapy	12 (6.8%)
Chemotherapy alone	77 (43.5%)
Radiotherapy alone	31 (17.5%)
Radiotherapy + chemotherapy	57 (32.2%)
With Rituximab	39 (22.0%)
CHOP/CHOP-like chemotherapy (*N* = 134)	104 (77.6%)
Clinical outcomes	
No evidence of disease	143 (80.8%)
Alive with disease	10 (5.7%)
Die of the disease	19 (10.7%)
Die of the other disease	5 (2.8%)

Abbreviations: *LDH* lactate dehydrogenase, *DLBCL* diffuse large B-cell lymphoma, *MALT* Mucosa Associated Lymphoid Tissue, *NHL* Non-Hodgkin lymphoma, *CHOP* cyclophosphamide, hydroxydaunorubicin, vincristine, and prednisone

CDS was performed in 59 (33.3%) patients. The H/BSO or radical hysterectomy for suspected primary cervical cancer was the most common surgical method, and radical trachelectomy was applied to patients who desired to preserve their fertility. Regarding the chemotherapy and/or radiotherapy as the predominant therapeutic option, 93.2% of patients received at least one therapy, while 57 patients experienced a combination therapy. Besides, CHOP or CHOP-like chemotherapy was the most frequently used regimen, accounting for 77.6% of patients treated with chemotherapy. In addition, rituximab was prescribed for 39 patients (Table 2).

**Fig. 2:**
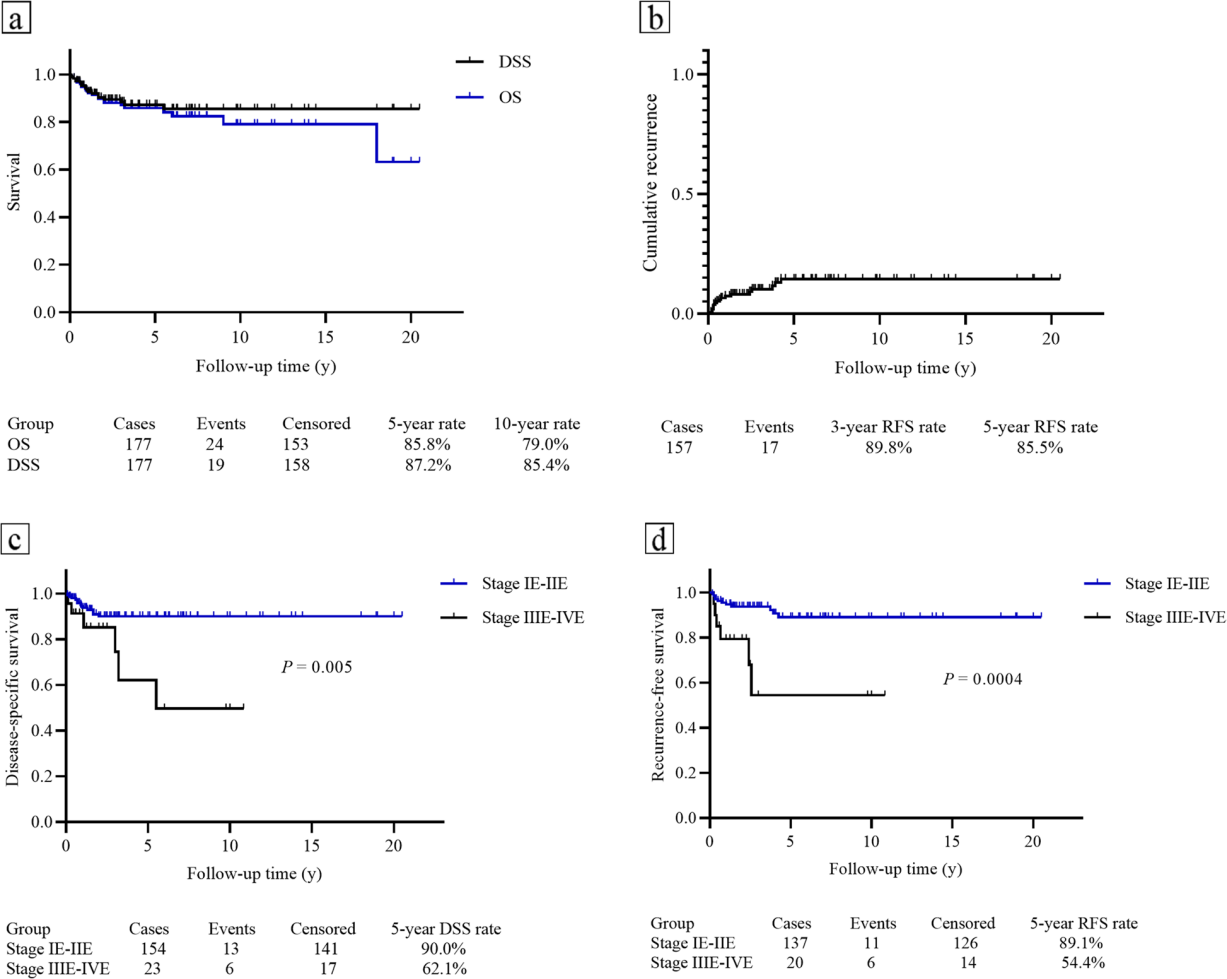
The Kaplan-Meier (log-rank test) curves for patients with PCL. **a** The overall survival (OS) and disease-specific survival (DSS) rates in this study. **b** The cumulative recurrence in patients with PCL. **c** Significant differences in DSS rates divided by Ann Arbor stage (IE-IIE vs. IIIE-IVE). **d** Patients with early-stage disease showed significantly better RFS compared with those with advanced stage disease

After a median follow-up time of 2.9 years, 80.8% of these patients achieved no evidence of disease, and the other 10 patients were alive with disease. Only 24 deaths occurred, with 5-year and 10-year OS rates of 85.8 and 79.0%, respectively (Fig. 2a). Among the 24 patients who died during follow-up, 19 deaths were related to cancer. The 5-year and 10-year DSS rates were 87.2 and 85.4%, respectively (Fig. 2a). Patients who had early disease stage (IE-IIE) showed significant better DSS and RFS than advanced stage (IIIE – IVE) (Fig. 2c and d). One hundred and fifty-seven patients achieved CR after initial treatment, in which 17 patients experienced the disease recurrence. The prognosis was extremely poor after recurrence, such that 9 of 17 patients died of the disease, and only 4 patients achieved no evidence of disease even after the salvage treatment. The 5-year RFS rate was 85.5% (Fig. 2b).

#### Subgroup analysis of patients with DLBCL

**Fig. 3:**
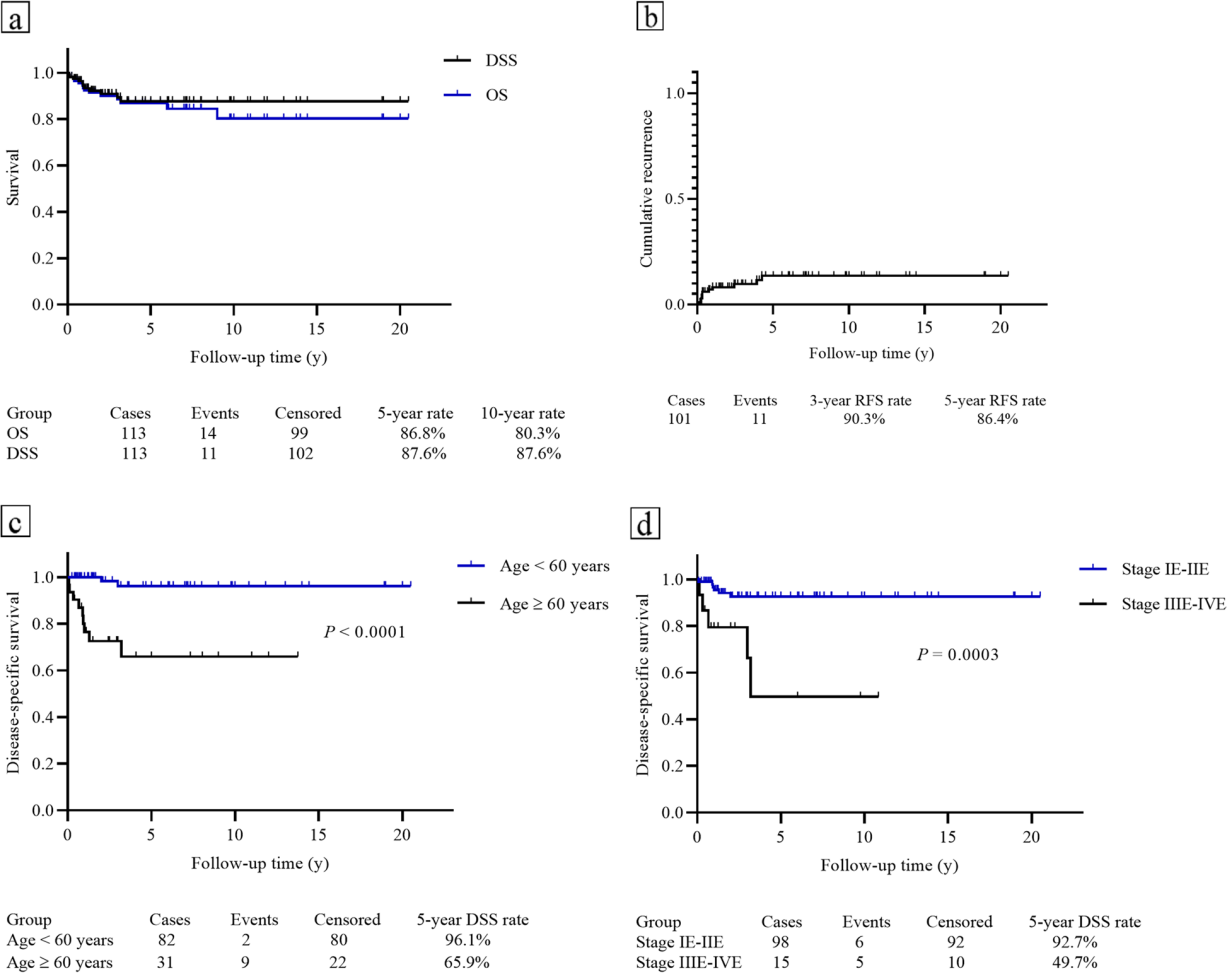
Survival curves for PCL patients of DLBCL subtypes. **a** The OS and DSS in this subgroup. **b** The 3-year and 5-year RFS rate. **c** The significant difference of DSS in patients who aged ≥60 and < 60 years. **d** Patients with Ann Arbor stage IE-IIE showed significantly better DSS compared with those with Ann Arbor stage IIIE-IVE

The clinical characteristics, treatment, and survival outcomes of patients with DLBCL were listed in Table 3. The 5-year and 10-year OS rate were 86.8 and 80.3%, respectively; with a 10-year DSS rate of 87.6% (Fig. 3a). A total of 101 patients obtained CR after initial treatment but 11 of them had disease recurrence and the 5-year RFS rate was 86.4% (Fig. 3b).

**Table 3:** Clinical characteristics of patients with primary cervical lymphoma of DLBCL subtype (*N* = 113)

Clinical characteristics	Number (Percentile)
Age (Mean/Median, y)	49.5 ± 16.3/49.0 (20–85)
Time of follow-up (Mean/Median, y)	4.7/3.0 (0.08–20.50)
Elevated LDH (*N* = 20)	12 (60%)
Ann Arbor Stage	
IE	73 (64.6%)
IIE	25 (22.1%)
IIIE	4 (3.5%)
IVE	11 (9.7%)
Cancer-directed surgery (CDS)	31 (27.4%)
Chemotherapy/Radiotherapy details	
No chemotherapy or radiotherapy	6 (5.3%)
Chemotherapy alone	57 (50.4%)
CHOP	17
CHOP + CDS	9
R-CHOP	13
R-CHOP + CDS	7
Radiotherapy alone	13 (11.5%)
Radiotherapy + chemotherapy	37 (32.7%)
Radiotherapy + CHOP	16
Radiotherapy + CHOP + CDS	1
Radiotherapy + R-CHOP	13
Radiotherapy + R-CHOP + CDS	1
Clinical outcomes	
No evidence of disease	93 (82.3%)
Alive with disease	6 (5.3%)
Die of the disease	11 (9.7%)
Die of the other disease	3 (2.7%)

Abbreviations: *LDH* lactate dehydrogenase, *DLBCL* diffuse large B-cell lymphoma, *(R)-CHOP* (Rituximab)-cyclophosphamide, hydroxydaunorubicin, vincristine, and prednisone

The potential risk factors for OS, DSS, and RFS identified by univariate and multivariate analyses are summarized in Supplementary Table S2. Age over 60 years and an advanced Ann Arbor stage were associated with poor DSS in the univariate analysis (Fig. 3c-d). These two factors and chemotherapy/radiotherapy were subsequently included in the multivariate analysis. Age ≥ 60 years (Odds ratio [OR]: 23.015, 95% Confidence Interval [CI]: 3.857–137.324, *P* = 0.001), and stage IIIE-IVE (OR: 4.056, 95% CI: 1.137–14.469, *P* = 0.031) remained statistically significant. Moreover, chemotherapy/radiotherapy (*P* = 0.049) significantly improved the DSS compared with those who did not receive any such interventions. However, combined chemotherapy and radiotherapy showed no significant difference in therapeutic effect when compared with chemotherapy or radiotherapy alone (*P* = 0.241).

Similarly, multivariate Cox regression analysis revealed that patients with age ≥ 60 years (OR: 26.324, 95%CI: 5.090–136.144, *P* < 0.001) or stage IIIE-IVE (OR: 4.219, 95%CI: 1.314–13.551, *P* = 0.016) suffered from a poor OS. Chemotherapy and/or radiotherapy (*P* = 0.008) improved the OS when compared with those who received no such therapy. However, only chemotherapy and/or radiotherapy (*P* = 0.003) manifested predictive potential in RFS (Fig. S2).

Moreover, no potential risk factor was identified in patients with DLBCL of Ann Arbor stage IE (Table S3).

## Discussion

Our current study presented one of the largest cohorts emphasizing on the survival outcomes and related prognostic factors in patients with PCL. Furthermore, we firstly evaluated the prognostic predictors for OS, DSS, and RFS in patients with DLBCL subtype. This may help to improve the disease’s management through adding novel perspectives.

This is the first study showing satisfactory OS and DSS rates in patients with PCL. Previous several research on the primary lymphoma of female genital tract included lymphoma of the uterus corpus, vulvar-vagina, and ovary [4, 5, 11, 12]. In 2016, Hilal et al. reported an OS rate of 81% in 61 cases but they did not clearly point out the exact timeline survival outcomes [3]. Similarly, another study by Nasioudis et al. revealed a 5-year DSS rates of 75.2% in a cohort of 697 patients [5]. However, only 129 patients presented lymphoma of the cervix and they did not conduct subgroup analysis of patients with DLBCL. Our current research further extended these findings, presenting respective higher rates of 5-year OS and DSS as 85.8 and 87.2%. The 5-year survival outcomes in this overall cohort were much better than that of both primary nodal lymphoma and extranodal lymphoma that the 5-year OS and DSS was about 60 to 70% [14, 15]. This may be attributed to the fact that most patients in our cohort were in the early stage of the disease and more than 60% of them had the DLBCL subtype. Our study demonstrated both the 5-year OS and DSS of stage IE DLBCL were exceeding 90%, comparable with the 5-year OS rates in patients with extranodal stage-I DLBCL [15]. Nonetheless, since specific information to determine the FIGO stage were unavailable because most cases were reviewed, it might be difficult to compare the survival outcomes with primary cervical cancer due to the different staging system.

There remains no practical risk stratification system for the PCL. We found that age over 60 years and Ann Arbor stage III-IVE were the two major factors predicting significantly worse OS and DSS rates in patients with PCL of DLBCL subtype. It is reasonable to consider that elderly patients have much less tolerance for rigorous treatment interventions and advanced-stage patients are associated with a higher tumor burden. Molecular characteristics such as ABC subtype, BCL2 expression, or cytogenetic complexity that associated with poor prognosis increased with age at diagnosis in patients with DLBCL [16]. Moreover, age and Ann Arbor stage are two factors in the prognostic predictive model of aggressive NHL treated with CHOP-like chemotherapy [17]. Compared with the previous study by Nasioudis et al. [5], we set modified cut-off values for the age and Ann Arbor stage according to the International Prognostic Index (IPI). In their study, the age cut-off was set at 55 years and patients were divided into the stage I and stage II-IV disease, which might have factitiously underestimated the survival outcomes in some patients. Furthermore, we detected no potential prognostic predictors in patients with DLBCL of stage IE and the 5-year DSS rate was exceeding 90% in patients with stage IE-IIE, indicating that stage IIE maybe a better cut-off to predict survival outcomes.

Relapse risk after initial treatment varies in PCL patients that published studies have shown the range of recurrence from 2 to 19% [3, 6, 10]. Our study demonstrated a comparable 5-year cumulative recurrent rate of 14.5% in overall population and 5-year RFS of 86.4% in DLBCL subtype. However, more than 70% of cases experiencing a relapse in less than 2 years after initial treatment. Furthermore, disease recurrence significantly impaired the survival outcomes in patients with PCL in this cohort, as more than half of them died of recurring PCL. Besides, in four patients with CNS relapse, 75% of them died, indicating the lethality of CNS recurrence. Indeed, the CNS recurrence in lymphoma is one of the most devastating complications [18] and primary DLBCL of the female genital tract could associate with a high risk of CNS recurrence [19]. This emphasized the importance of proper treatment to lower the recurrence rate.

Although there is no consensus on the management of PCL, prior studies have discussed the role of different therapeutic methods but mainly restricted to narrative descriptions [3, 5, 6, 12]. We strongly recommend that treatment options for PCL should be in accordance with the guidelines for extranodal lymphoma. Chemotherapy and/or radiotherapy is the cornerstone in treating extranodal lymphoma, and the specific therapeutic scheme and dose depends on the corresponding pathological subtypes [20]. The addition of rituximab or other monoclonal antibodies can significantly enhance the efficacy of the traditional chemotherapy of radiotherapy, and plays an important role in maintenances treatment or relapsed diseases [21, 22]. In our study, CHOP or CHOP-like chemotherapy occupied the largest proportion of treatments; 39 patients were treated combined with rituximab (R-CHOP). This may attribute to the DLBCL as the predominant subtype and R-CHOP is the first-line mainstay treatment [23]. Our research revealed that a combination of radiotherapy and chemotherapy tended to better improve the patients’ RFS, OS, and DSS compared with those treated with chemotherapy or radiotherapy alone. Moreover, one-third of patients underwent CDS but neither could improve the all-cause mortality/disease-specific death nor lower the probability of recurrence. Likewise, Nasioudis et al. also found no association between CDS and OS/DSS in a large cohort of 697 cases [5]. However, currently there is no distinct imaging characteristic that can facilitate distinguishing between primary cervical cancer and cervical lymphoma [12, 24], comprehensive evaluation, especially through biopsy of the lesion to confirm diagnosis before CDS is strongly recommended. Radical surgery should be avoided when the diagnosis is determined.

Several limitations that should be underlined. The heterogeneity of this study could not be neglected since most cases were retrieved from the literature. Besides, some important information had been missing in reviewed articles, such as the ECOG performance status. Prognostic predictors were unavailable in other pathologic subtypes due to extremely rarity. In addition, a small proportion of DLBCL received rituximab-based treatment, the current standard treatment of DLBCL. Moreover, we excluded cases reported in letters and non-English literature, and the median follow-up time was relatively short, which may bias the results. Further research to optimize the management of PCL is needed.

## Conclusion

Patients with PCL in early stage have satisfactory survival outcomes, the advanced Ann Arbor stage and age ≥ 60 years are two major factors predicting poor prognosis in patients with DLBCL subtype. Combined chemotherapy and radiotherapy or alone in accordance with the clinical guidelines of extranodal lymphoma is recommended, while the cancer-directed surgery should be avoided.

## Supplementary Information

**Additional file 1: Supplementary fig. S1.** The inclusion process summarized in the PRISMA flow diagram.

**Additional file 2: Table S1.** Database of patients with primary lymphoma in this study.

**Additional file 3: Table S2a.** Univariate and multivariate analysis of OS in DLBCL population. **Table S2b.** Univariate and multivariate analysis of DSS in DLBCL population. **Table S2c.** Univariate and multivariate analysis of RFS in DLBCL population (*N* = 101).

**Additional file 4: Supplementary fig. S2.** The Kaplan-Meier (log-rank test) curves of OS/RFS for patients with cervical DLBCL. (**a**) Age ≥ 60 years and < 60 years. (**b**) Ann Arbor stage IE-IIE versus IIIE-IVE. (**c**) Impact of single combine treatment on OS. (**d**) Impact of single or combined treatment on RFS.

**Additional file 5: Table S3a.** Univariate and multivariate analysis of OS in DLBCL population (Ann Arbor stage IE). **Table S3b.** Univariate and multivariate analysis of DSS in DLBCL population (Ann Arbor stage IE). **Table S3c.** Univariate and multivariate analysis of RFS in DLBCL population (Ann Arbor stage IE).

## Data Availability

All data generated or analyzed during this study are included in this published article and the supplementary information files. The datasets used and/or analyzed during the current study can be obtained from the corresponding author upon reasonable request.

## Abbreviations

PCL: Primary cervical lymphoma
NHL: Non-Hodgkin’s lymphoma
DLBCL: Diffuse large B-cell lymphoma
R-CHOP: Rituximab plus cyclophosphamide, doxorubicin, vincristine, and prednisone.
