# Supplementary figures and images for: Advanced Ann Arbor stage and age over 60 years as prognostic predictors in patients with primary cervical lymphoma: a retrospective cohort study and systematic review
Source: BMC Cancer. 2023 Jan 27;23:95. doi: 10.1186/s12885-023-10548-4 (PMC9881271; doi:10.1186/s12885-023-10548-4)

Supplementary figure S1. The inclusion process summarized in the PRISMA flow diagram.

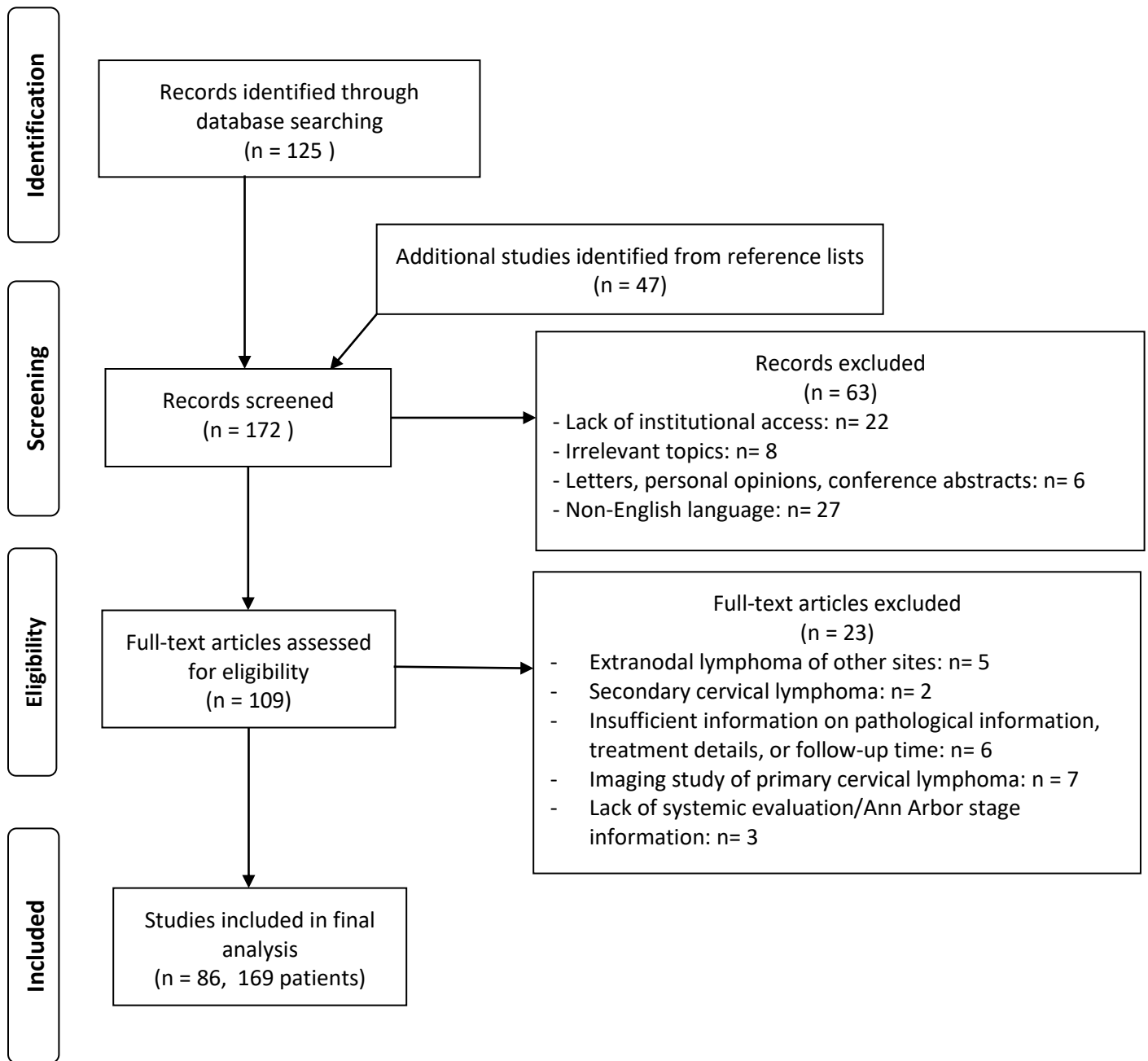

Supplement: Supplementary file 1 — Additional file 1: Supplementary fig. S1. The inclusion process summarized in the PRISMA flow diagram. [file 12885_2023_10548_MOESM1_ESM.pdf]

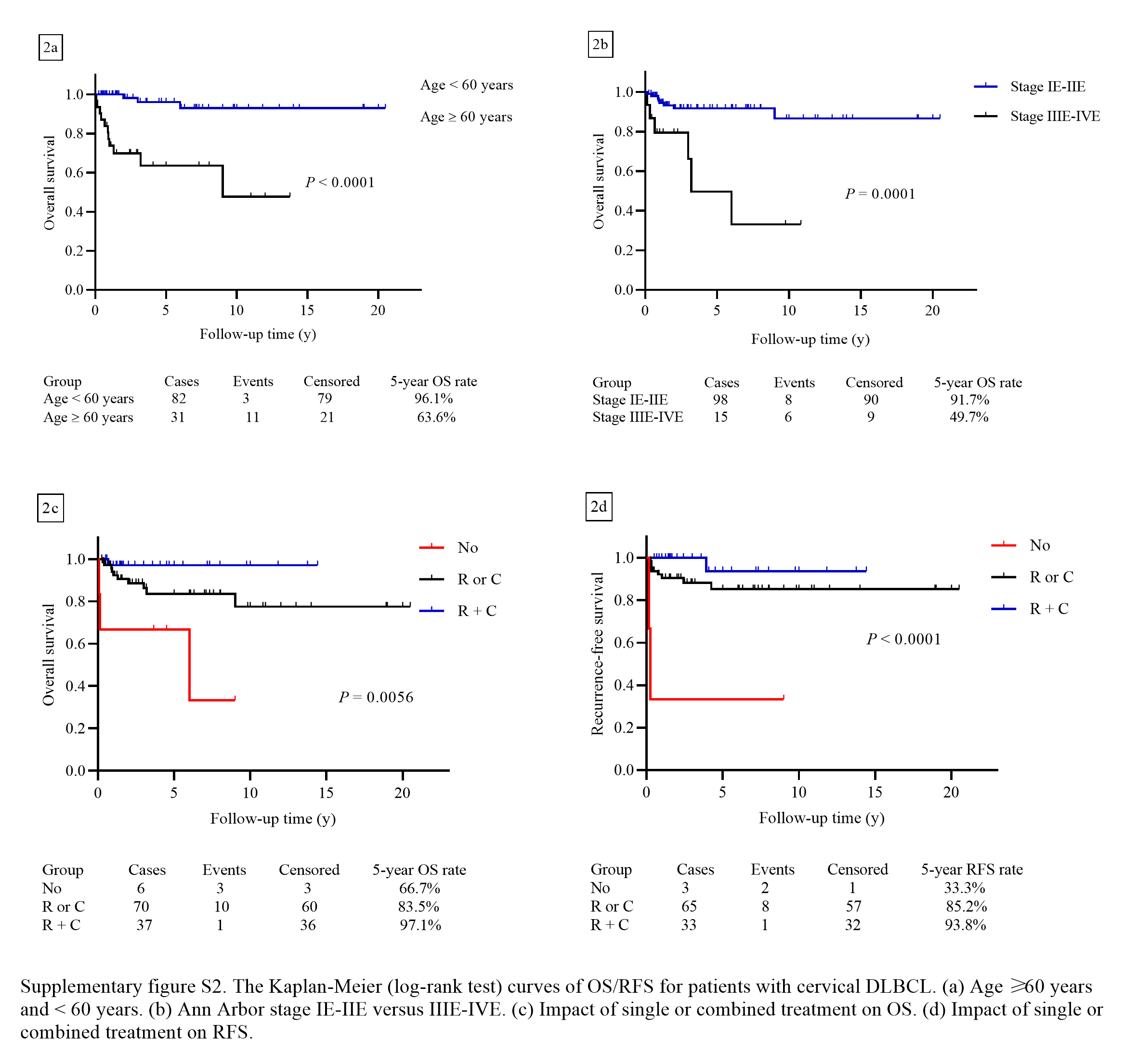

Supplement: Supplementary file 4 — Additional file 4: Supplementary fig. S2. The Kaplan-Meier (log-rank test) curves of OS/RFS for patients with cervical DLBCL. (a) Age ≥ 60 years and < 60 years. (b) Ann Arbor stage IE-IIE versus IIIE-IVE. (c) Impact of single combine treatment on OS. (d) Impact of single or combined treatment on RFS. [file 12885_2023_10548_MOESM4_ESM.tif]
