# Supplementary material for: Advanced Ann Arbor stage and age over 60 years as prognostic predictors in patients with primary cervical lymphoma: a retrospective cohort study and systematic review
Source: BMC Cancer. 2023 Jan 27;23:95. doi: 10.1186/s12885-023-10548-4 (PMC9881271; doi:10.1186/s12885-023-10548-4)
Supplement: Supplementary file 2 — Additional file 2: Table S1. Database of patients with primary lymphoma in this study. [file 12885_2023_10548_MOESM2_ESM.docx]

Table S1. Database of patients with primary lymphoma in this study..

| Reference | No. | Age  (y) | Stage | Pathology  subtypes | CDS | CDS options; involvement sites | C | Chemotherapy options | R | Re | RFS (y) | Recurrence | Status | OS (y) | DSS |
| --- | --- | --- | --- | --- | --- | --- | --- | --- | --- | --- | --- | --- | --- | --- | --- |
| Cases in our hospital | 1 | 66 | IVAE | 1 | 0 | Cervix, uterus (extensive involved); pelvic lymph nodes, bladder | 1 | R-CHOP*6 | 1 | NA |  |  | 3 | 0.67 | 1 |
|  | 2 | 72 | IE | 1 | 0 |  | 1 | R-CHOP*5 | 0 | 0 | 8.04 |  | 0 | 8.04 | 0 |
|  | 3 | 56 | IVAE | 1 | 1 | TAH+BSO+pelvic and para-aortic LN resection  Bone marrow | 1 | R-EPOCH*9, intrathecal injection of Ara-C + DXM | 0 | 0 | 1 |  | 0 | 1 | 0 |
|  | 4 | 62 | IVAE | 1 | 0 | Bladder, extensive cervix, and uterus involvement | 0 |  | 0 | NA |  |  | 3 | 0.12 | 1 |
|  | 5 | 31 | IVE | 4 | 0 | Bone | 1 | R-hyperCVAD*3, R-MA*2,CVP-R*1 | 0 | 1 | 0.42 | T11, L2 lumbar | 1 | 0.6 | 0 |
|  | 6 | 58 | IVAE | 1 | 0 | Liver (extensive involvement) | 1 | R-CHOP*8 | 0 | 0 | 2.25 | extenvisve LN involved | 0 | 2.25 | 0 |
|  | 7 | 71 | IVAE | 1 | 0 | Lung (extensive involvement) | 1 | R-CHOP*6 (4 with Ara-C +DXM intrathecal injection) | 0 | NA |  |  | 3 | 3.2 | 1 |
|  | 8 | 58 | IE | 1 | 0 |  | 1 | R-CHOP*8 （Ara-C+MTX intrathecal injection*4） | 0 | 0 | 7.33 |  | 0 | 7.33 | 0 |
| Komaki et al. (1984) ^1^ | 9 | 43 | IIE | 1 | 0 | Parametrium, pelvic lymph nodes | 0 |  | 1 | 1 | 4.25 | right kidney | 0 | 13 | 0 |
|  | 10 | 38 | IIE | 8 | 0 | Parametrium | 0 |  | 1 | 0 | 3 |  | 0 | 3 | 0 |
|  | 11 | 38 | IIE | 1 | 0 | Left iliac lymph nodes | 0 |  | 1 | 0 | 7 |  | 0 | 7 | 0 |
| Bär et al. (1986)^2^ | 12 | 66 | IE | 1 | 1 | TH+BSO+parametrectomy+pelvic LN resection | 1 | CHOP*6 (CTX+Hydroxydaunorubicin+VCR+Prednisone） | 0 | 1 | 0.33 | CNS | 3 | 1.3 | 1 |
| Miketic et al. (1988)^3^ | 13 | 74 | IE | 1 | 0 |  | 0 |  | 1 | 0 | 1 |  | 0 | 1 | 0 |
|  | 14 | 56 | IE | 8 | 1 | TAH+BSO | 0 |  | 1 | 0 | 1 |  | 0 | 1 | 0 |
| Strang et al. (1988)^4^ | 15 | 77 | IE | 8 | 0 |  | 1 | COP*7 (CTX+VCR+prednisolone) | 1 | 0 | 3 |  | 0 | 3 | 0 |
| Johnston et al. (1989)^5^ | 16 | 22 | IE | 6 | 0 |  | 1 | MACOP-B*12weeks course (MTX+ADM+CTX+VCR+Prednisone+BLM) | 1 | 0 | 2.75 |  | 0 | 2.75 | 0 |
| Sandvei et al. (1990)^6^ | 17 | 22 | IE | 8 | 0 |  | 1 | CHOP for 6 course | 0 | 0 | 5.5 |  | 0 | 5.5 | 0 |
| Muntz et al. (1991)^7^ | 18 | 30 | IE | 1 | 1 | radical TAH+BSO+pelvic LN resection | 0 |  | 1 | 0 | 10 |  | 0 | 10 | 0 |
|  | 19 | 73 | IE | 1 | 0 |  | 0 |  | 1 | 0 | 5 |  | 0 | 5 | 0 |
|  | 20 | 23 | IE | 1 | 0 |  | 0 |  | 1 | 0 | 5 |  | 0 | 5 | 0 |
|  | 21 | 73 | IE | 8 | 0 | TAH+BSO+pelvic LN resection for uterine myoma | 0 |  | 1 | 0 | 4.5 |  | 0 | 4.5 | 0 |
|  | 22 | 57 | IE | 2 | 1 | TAH+BSO | 0 |  | 1 | 0 | 4.5 |  | 0 | 4.5 | 0 |
| Harris et al. (1984)^8^ | 23 | 49 | IE | 2 | 1 | TAH+BSO | 0 |  | 0 | 0 | 14 |  | 0 | 14 | 0 |
|  | 24 | 43 | IE | 2 | 1 | TAH+BSO | 0 |  | 1 | 0 | 13 |  | 0 | 13 | 0 |
|  | 25 | 24 | IE | 1 | 0 |  | 0 |  | 1 | 0 | 9 |  | 0 | 9 | 0 |
|  | 26 | 20 | IE | 1 | 0 |  | 0 |  | 1 | 0 | 6.3 |  | 0 | 6.3 | 0 |
|  | 27 | 38 | IE | 8 | 1 | TAH | 0 |  | 1 | 0 | 11 |  | 0 | 11 | 0 |
|  | 28 | 49 | IE | 2 | 1 | TAH+BSO | 0 |  | 0 | 0 | 4 |  | 0 | 4 | 0 |
|  | 29 | 26 | IE | 2 | 1 | TAH+BSO | 0 |  | 1 | 0 | 3.2 |  | 0 | 3.2 | 0 |
|  | 30 | 50 | IE | 2 | 1 | TAH+BSO | 0 |  | 1 | 0 | 2 |  | 0 | 2 | 0 |
|  | 31 | 34 | IE | 1 | 1 | Radical TAH+BSO+pelvic LN resection | 0 |  | 0 | 0 | 9 |  | 0 | 9 | 0 |
|  | 32 | 23 | IE | 1 | 1 | TAH+BSO | 0 |  | 1 | 0 | 7.1 |  | 0 | 7.1 | 0 |
|  | 33 | 30 | IE | 1 | 1 | radical TAH+BSO+pelvic LN resection | 0 |  | 1 | 0 | 2.1 |  | 0 | 2.1 | 0 |
|  | 34 | 80 | IE | 1 | 1 | TAH+BSO | 1 | COP | 0 | NA |  |  | 3 | 0.9 | 1 |
| Broekmans et al. (1983)^9^ | 35 | 45 | IE | 6 | 0 |  | 1 | CHOP-MTX*6 | 0 | 0 | 3 |  | 0 | 3 | 0 |
| Kasales et al. (1993)^10^ | 36 | 20 | IE | 8 | 0 |  | 1 | CHOP*5 | 1 | 0 | 0.67 |  | 0 | 0.67 | 0 |
|  | 37 | 31 | IE | 8 | 0 |  | 1 | ProMACE+CytoBOM | 0 | 0 | 3 |  | 0 | 3 | 0 |
|  | 38 | 53 | IE | 8 | 1 | TAH+BSO | 1 | CHOP | 1 | 0 | 6 |  | 0 | 6 | 0 |
| Awwad et al. (1994)^11^ | 39 | 27 | IE | 8 | 0 |  | 0 |  | 1 | 1 | 0.5 | skull, CNS | 3 | 0.6 | 1 |
| Kuo et al. (1994)^12^ | 40 | 40 | IE | 1 | 1 | extended TH+LSO+pelvic LN resection | 1 | COP-BLAM*6(one course NAC) | 0 | 0 | 2 |  | 0 | 2 | 0 |
| Makarewicz et al. (1995)^13^ | 41 | 37 | IE | 1 | 0 |  | 1 | COP*3 | 1 | 0 | 8 |  | 0 | 8 | 0 |
|  | 42 | 33 | IE | 1 | 1 | TAH+BSO+pelvic LN resection | 0 |  | 0 | 1 | 0.16 | vagina and parametria | 0 | 3.66 | 0 |
|  | 43 | 65 | IE | 6 | 0 |  | 1 | COP*6 | 1 | 0 | 3 |  | 0 | 3 | 0 |
| Patsner et al. (1995)^14^ | 44 | 38 | IE | 8 | 1 | radical hysterectomy | 1 | CHOP*6 | 1 | 0 | 3 |  | 0 | 3 | 0 |
| Stroh et al. (1995)^15^ | 45 | 53 | IE | 1 | 0 |  | 1 | CHOP-BLM | 1 | 0 | 14.42 |  | 0 | 14.42 | 0 |
|  | 46 | 64 | IE | 1 | 0 |  | 1 | CHOP-BLM | 1 | 0 | 13.75 |  | 0 | 13.75 | 0 |
|  | 47 | 39 | IIE | 1 | 0 | Pelvic lymph nodes, vagina, parametrium | 1 | CHOP-BLM | 1 | 0 | 11.83 |  | 0 | 11.83 | 0 |
|  | 48 | 35 | IIIEB | 1 | 0 | Inguinal and axillary lymph nodes | 1 | CHOP-BLM | 1 | 0 | 9.75 |  | 0 | 9.75 | 0 |
|  | 49 | 73 | IVA | 1 | 0 | Left saphenous vein | 1 | CHOP-BLM | 0 | 1 | 0.33 | Multi-organ | 3 | 0.33 | 1 |
|  | 50 | 22 | IVB | 8 | 0 | Right breast, liver | 1 | MCOAP,HOAP-BLM,CEMO | 0 | 1 | 0.67 | epidural, bone, marrow | 3 | 1.08 | 1 |
|  | 51 | 69 | IVB | 8 | 0 | Spleen, liver, femoral, axillary, and inguinal lymph nodes | 1 | CHOP-BLM, INF | 0 | 1 | 2.58 | bone marrow | 3 | 5.5 | 1 |
|  | 52 | 64 | IE | 1 | 0 |  | 1 | CHOP-BLM | 0 | 1 | 0.33 | pelvic, retroperitoneal, mesenteric LN | 3 | 0.92 | 1 |
|  | 53 | 66 | IE | 1 | 0 |  | 1 | CHOP | 1 | 0 | 5 |  | 0 | 5 | 0 |
|  | 54 | 57 | IIE | 1 | 0 | Parametrium | 1 | CHOP-BLM, CMED | 1 | 0 | 5.58 |  | 0 | 5.58 | 0 |
|  | 55 | 49 | IIE | 8 | 0 | Parametrium | 1 | ASAP, BACOS,MINE | 1 | 0 | 3.08 |  | 0 | 3.08 | 0 |
|  | 56 | 62 | IIE | 2 | 0 | Pelvic lymph nodes | 1 | CHOP-BLM | 1 | 0 | 1.5 |  | 0 | 1.5 | 0 |
|  | 57 | 67 | IE | 1 | 0 |  | 1 | ASHAP,MBACOS,MINE | 1 | NA |  |  | 1 | 1.5 | 0 |
|  | 58 | 49 | IIE | 1 | 0 | Parametrium | 1 | CHOP-BLM,CMED | 1 | 0 | 7.17 |  | 0 | 7.17 | 0 |
|  | 59 | 78 | IIE | 1 | 0 | Inguinal lymph nodes | 1 | CHOP-BLM,CMED | 1 | 0 | 7.33 |  | 0 | 7.33 | 0 |
|  | 60 | 46 | IIE | 8 | 0 | Anterior sacral lymph nodes | 1 | CHOP-BLM | 1 | 0 | 1 |  | 0 | 1 | 0 |
| AI Talib et al. (1996)^16^ | 61 | 45 | IE | 1 | 0 |  | 1 | unspecified | 0 | 0 | 2 |  | 0 | 2 | 0 |
|  | 62 | 22 | IE | 1 | 0 |  | 1 | unspecified | 0 | 0 | 0.75 |  | 0 | 0.75 | 0 |
| Alberti et al. (1996) ^17^ | 63 | 69 | IE | 6 | 1 | TAH+BSO | 0 |  | 0 | 0 | 1 |  | 0 | 1 | 0 |
| Chandy et al. (1998)^18^ | 64 | 50 | IE | 1 | 0 |  | 1 | CHOP*9 | 1 | 0 | 1.45 |  | 0 | 1.45 | 0 |
| Clarke et al. (1998)^19^ | 65 | 28 | IE | 6 | 0 |  | 1 | CHOP | 0 | 0 | 0.5 |  | 0 | 0.5 | 0 |
| Lee et al. (1998)^20^ | 66 | 67 | IE | 1 | 1 | TAH+BSO+pelvic LN resection | 0 |  | 1 | 0 | 12 |  | 0 | 12 | 0 |
|  | 67 | 65 | IE | 1 | 1 | TAH+BSO+pelvic and paraortic LN resection | 0 |  | 1 | 0 | 11 |  | 0 | 11 | 0 |
| Nasu et al. (1998)^21^ | 68 | 64 | IE | 1 | 0 |  | 1 | CHOP*10 | 0 | 0 | 1.5 |  | 0 | 1.5 | 0 |
| Sungurtekin et al. (1998)^22^ | 69 | 47 | IV | 1 | 1 | sTAH+BSO; extensive cervix, uterus involvement, bilateral ovary | 1 | COP-MTX | 0 | 0 | 0.45 |  | 0 | 0.45 | 0 |
| Pelstring et al. (1991)^23^ | 70 | 40 | IE | 5 | 1 | Hysterectomy | 0 |  | 1 | 0 | 0.25 |  | 0 | 0.25 | 0 |
| Rossi et al. (2001)^24^ | 71 | 46 | IV | 5 | 1 | TAH+BSO; bone marrow | 1 | ProMACE+CytoBOM*5 | 0 | 0 | 2 |  | 0 | 2 | 0 |
| Yokoyama et al. (2001)^25^ | 72 | 55 | IIE | 1 | 0 | Pelvic LN | 1 | CHOP | 1 | 0 | 1 |  | 0 | 1 | 0 |
| Au et al. (2003)^26^ | 73 | 45 | IIE | 1 | 0 | Pelvic LN | 1 | (MTX, BLM, ADM, VCR, CTX, DXM)*6 | 0 | 0 | 8 |  | 0 | 8 | 0 |
| Szantho et al. (2003)^27^ | 74 | 56 | IE | 1 | 1 | Radical TAH+BSO+pelvic LN resection | 1 | CHOP*6 | 0 | 0 | 5 |  | 0 | 5 | 0 |
| Chan et al. (2005)^28^ | 75 | 62 | IE | 8 | 1 | H/BSO+pelvic lymphadenectomy | 0 |  | 1 | 0 | 1 |  | 0 | 1 | 0 |
| All B-cell NHL | 76 | 40 | IV | 1 | 1 | Trachelectomy+pelvic lymphadenectomy | 0 |  | 0 | 1 | 0.25 | liver and spleen | 3 | 6 | 0 |
|  | 77 | 41 | IIIEB | 1 | 0 |  | 1 | CHOP*8 | 0 | 1 | 2.42 | abdominal retroperitoneal lymph nodes | 3 | 3 | 1 |
|  | 78 | 49 | IVE | 8 | 0 |  | 1 | CHOP*8 | 1 | 0 | 10 |  | 0 | 10 | 0 |
|  | 79 | 76 | IE | 1 | 1 | H/BSO+pelvic lymphadenectomy | 1 | CHOP*9 | 0 | 0 | 2.5 |  | 0 | 2.5 | 0 |
|  | 80 | 52 | IVEB | 8 | 0 |  | 1 | CHOP | 0 | 0 | 1 |  | 0 | 1 | 0 |
| Perren et al. (1992)^29^ | 81 | 54 | IIE | 8 | 1 | nodal biopsy and splenectomy  parametrium | 0 |  | 1 | 0 | 18 |  | 3 | 18 | 0 |
|  | 82 | 47 | IIEB | 8 | 0 | Vagina | 1 | CHOP*6 | 0 | 0 | 6.25 |  | 0 | 6.25 | 0 |
|  | 83 | 53 | IE | 1 | 0 |  | 0 |  | 1 | 0 | 20 |  | 0 | 20 | 0 |
|  | 84 | 41 | IE | 8 | 0 |  | 1 | mustine procarbazine and prednisolone*3 | 1 | 0 | 8 |  | 0 | 8 | 0 |
| Vang et al. (2000)^30^ | 85 | 39 | IE | 1 | 0 |  | 1 |  | 1 | NA |  |  | 1 | 0.58 | 0 |
| All NHL | 86 | 46 | IE | 1 | 1 | TAH+BSO | 0 |  | 0 | NA |  |  | 1 | 4.5 | 0 |
|  | 87 | 57 | IIE | 1 | 0 |  | 1 |  | 1 | 0 | 10 |  | 0 | 10 | 0 |
|  | 88 | 57 | IIE | 1 | 0 |  | 1 |  | 1 | 0 | 5 |  | 0 | 5 | 0 |
|  | 89 | 61 | IIE | 2 | 1 | TAH+BSO | 1 |  | 1 | 0 | 6 |  | 0 | 6 | 0 |
|  | 90 | 65 | IE | 1 | 0 |  | 1 |  | 0 | 1 | 1 | multiple replase | 3 | 1 | 1 |
|  | 91 | 67 | IE | 1 | 0 |  | 1 |  | 0 | 0 | 9 |  | 3 | 9 | 0 |
|  | 92 | 67 | IE | 1 | 0 |  | 1 |  | 1 | 0 | 5 |  | 0 | 5 | 0 |
| Dursun et al. （2005^31^ | 93 | 51 | IE | 1 | 1 | TAH+BSO+pelvic and paraortic LN resection | 1 | CHOP*6 | 0 | 0 | 1.58 |  | 0 | 1.58 | 0 |
|  | 94 | 49 | IE | 2 | 0 |  | 1 | CHOP*6 | 0 | 0 | 1.83 |  | 0 | 1.83 | 0 |
| Dhimes et al. (1996) ^32^ | 95 | 69 | IE | 6 | 1 | TAH+BSO | 0 |  | 0 | 0 | 1 |  | 0 | 1 | 0 |
| Gutman et al. (1998)^33^ | 96 | 57 | IVB | 6 | 1 | TAH+BSO+pelvic and para-aortic LN resection | 1 | CHOP*6 | 0 | 0 | 2.5 |  | 0 | 2.5 | 0 |
| Bode et al. (2002)^34^ | 97 | 46 | IE | 2 | 0 |  | 1 | CEOP*3 | 1 | 0 | 0.83 |  | 0 | 0.83 | 0 |
| Pham et al. (2003) ^35^ | 98 | 36 | IE | 1 | 0 |  | 1 | CHOP*6 | 0 | 0 | 3.17 |  | 0 | 3.17 | 0 |
| Baxter et al. (2003)^36^ | 99 | 75 | IE | 2 | 0 |  | 1 | chlorambucil | 0 | NA |  |  | 1 | 0.5 | 0 |
| Kahlifa et al. (2003)^37^ | 100 | 32 | IE | 1 | 0 |  | 1 | CHOP*6 | 1 | 0 | 0.83 |  | 0 | 0.83 | 0 |
| Garavaglia et al. (2005)^38^ | 101 | 38 | IE | 1 | 0 |  | 1 | MACOP-B*6 | 0 | 0 | 10 |  | 0 | 10 | 0 |
|  | 102 | 38 | IIE | 1 | 1 | TAH+BSO+pelvic lymphadenectomy  Vaginal wall, right parametrial LN | 1 | MACOP-B, 12 weekly cycles | 0 | 0 | 7 |  | 0 | 7 | 0 |
|  | 103 | 35 | IIE | 1 | 1 | TAH  Pelvic lN | 1 | CHOP*4 | 0 | 0 | 6 |  | 0 | 6 | 0 |
| Heredia et al. (2005)^39^ | 104 | 32 | IE | 6 | 0 |  | 1 | CHOP*3 | 1 | 0 | 5.1 |  | 0 | 5.1 | 0 |
|  | 105 | 31 | IIE | 6 | 1 | Radical TAH+BSO+pelvic LN resection  Pelvic LN | 1 | mCHOP*4 | 1 | 0 | 1.3 |  | 0 | 1.3 | 0 |
| Kendrick et al. (2005)^40^ | 106 | 66 | IV | 6 | 1 | TAH+BSO+omentectomy  Extensive involved cervix, uterus | 1 | R-CHOP*8 | 0 | 0 | 1.5 |  | 0 | 1.5 | 0 |
|  | 107 | 47 | IE | 6 | 0 |  | 1 | R-CHOP*4 | 1 | 0 | 1.3 |  | 0 | 1.3 | 0 |
| Frey et al. (2006)^41^ | 108 | 35 | IIE | 1 | 1 | TAH  Pelvic LN | 1 | R-CHOP*4 | 0 | 0 | 3 |  | 0 | 3 | 0 |
|  | 109 | 56 | IIE | 1 | 1 | TAH+BSO  Pelvic LN | 1 | R-CHOP*8 | 0 | 1 | 0.75 | retroperitoneum | 0 | 1.25 | 0 |
|  | 110 | 49 | IIE | 1 | 1 | TAH+BSO+bilateral lymph node sampling  Right iliac LN | 1 | R-CHOP*6, R*6 for maintenance | 0 | 0 | 2.67 |  | 0 | 2.67 | 0 |
| Hariprasad et al. (2006)^42^ | 111 | 47 | IE | 1 | 0 |  | 1 | CHOP*3, COP*3 | 1 | 0 | 0.83 |  | 0 | 0.83 | 0 |
|  | 112 | 80 | IE | 1 | 0 |  | 1 | CHOP*6 | 0 | 0 | 1 |  | 0 | 1 | 0 |
| Leon et al. (2006)^43^ | 113 | 56 | IIE | 1 | 0 | Parametrial LN | 1 | CHOP*8 | 1 | 0 | 0.5 |  | 0 | 0.5 | 0 |
| Cohn et al. (2007)^44^ | 114 | 64 | IE | 1 | 0 |  | 1 | R-CHOP*5 | 0 | NA |  |  | 2 | 1.1 | 0 |
|  | 115 | 46 | IIE | 1 | 0 | Pelvic LN | 1 | R-CHOP*6 | 0 | 1 | 0.25 | CNS | 3 | 2 | 1 |
|  | 116 | 22 | IE | 1 | 0 |  | 1 | R-CHOP*6 | 1 | 0 |  |  | 0 | 1.2 | 0 |
| Korcum et al. (2007)^45^ | 117 | 67 | IE | 2 | 0 |  | 1 | CHOP*3 | 1 | 1 | 1.33 | neck nodal | 0 | 3.25 | 0 |
| Lorusso et al. (2007)^46^ | 118 | 29 | IE | 6 | 0 |  | 1 | CHOP*6 | 0 | 0 | 3 |  | 0 | 5 | 0 |
| Hamid et al. (2008)^47^ | 119 | 43 | IE | 1 | 0 |  | 1 | CHOP*6 | 0 | 0 | 0.5 |  | 0 | 0.5 | 0 |
| Hanprasertpong et al. (2008)^48^ | 120 | 25 | IE | 1 | 0 |  | 1 | CHOP*6 | 0 | 0 | 3 |  | 0 | 3 | 0 |
| Upanal et al. (2011)^49^ | 121 | 49 | IE | 1 | 0 |  | 1 | R-CHOP*6 | 1 | 0 | 1.67 |  | 0 | 1.67 | 0 |
|  | 122 | 51 | IIE | 1 | 0 | Pelvic (pre-sacral) LN | 1 | R-CHOP*6 | 1 | 0 | 1.58 |  | 0 | 1.58 | 0 |
| Parva et al. (2011)^50^ | 123 | 21 | IE | 1 | 0 |  | 1 | R-CHOP*6 | 0 | 0 | 6 |  | 0 | 6 | 0 |
| Ferreri et al. (2008) ^51^ | 124 | 29 | IE | 1 | 0 |  | 1 | R-CHOP*6 | 1 | 0 | 4.5 |  | 0 | 4.5 | 0 |
| Binesh et al. (2012)^52^ | 125 | 85 | IE | 1 | 0 |  | 1 | R-CHOP*3 | 0 | NA |  |  | 3 | 0.42 | 0 |
| Parnis et al. (2012)^53^ | 126 | 54 | IE | 1 | 0 |  | 1 | R-CHOP*6 | 1 | 0 | 1.42 |  | 0 | 1.42 | 0 |
| Groszmann et al. (2013)^54^ | 127 | 25 | IE | 1 | 0 |  | 1 | unspecified | 0 | 0 | 1 |  | 0 | 1 | 0 |
| Mandato et al. (2014)^55^ | 128 | 44 | IVE | 1 | 1 | radical hysterectomy  extensive involved cervix, upper vagina | 1 | R-CHOP*6 | 0 | 0 | 2 |  | 0 | 2 | 0 |
| Adachi et al. (2015)^56^ | 129 | 69 | IV | 1 | 0 | Bladder, pelvic LN, and bone marrow | 1 | R-CHOP*5, CHOP*1 | 0 | 0 | 3 |  | 0 | 3 | 0 |
| Li et al. (2015)^57^ | 130 | 43 | IE | 7 | 1 | hysterectomy | 1 | ABVD (doxorubicin, bleomycin, vinblastine and dacarbazine) | 0 | NA |  |  | 1 | 1 | 0 |
| Wang et al. (2015)^58^ | 131 | 54 | IE | 3 | 1 | TAH+BSO | 0 |  | 0 | NA |  |  | 3 | 0.2 | 1 |
| Dobrosavljevic et al. (2016)^59^ | 132 | 37 | IE | 2 | 1 | H/BSO | 0 |  | 0 | 0 | 1 |  | 0 | 1 | 0 |
| Regalo et al. (2016)^60^ | 133 | 40 | IIE | 2 | 0 | Pelvic LN | 1 | R-CHOP*1, R-COP*8 | 0 | 1 | 3.75 | cervix, vagina | 1 | 4 | 0 |
| Zhou et al. (2016)^61^ | 134 | 31 | IE | 1 | 0 |  | 1 | R-CHOP*6 | 0 | 0 | 0.4 |  | 0 | 0.4 | 0 |
| Cubo et al. (2017)^62^ | 135 | 51 | IE | 1 | 0 |  | 1 | R-CHOP*6 | 0 | 0 | 2 |  | 0 | 2 | 0 |
| Kosari et al. (2017)^63^ | 136 | 49 | IE | 3 | 0 |  | 1 | CHOP | 1 | NA |  |  | 3 | 1.67 | 1 |
| Omori et al. (2016)^64^ | 137 | 65 | IIE | 3 | 0 | Pelvic (Left external iliac) LN | 1 | CHOP*6 | 0 | NA |  |  | 3 | 0.33 | 1 |
| Roberts et al. (2018)^65^ | 138 | 55 | IE | 1 | 1 | Hysterectomy with BSO | 1 | R-CHOP*3 | 0 | 0 | 3 |  | 0 | 3 | 0 |
| Gui et al. (2019)^66^ | 139 | 65 | IE | 1 | 1 | adnexectomy and resection of pelvic LN | 1 | R-CHOP*8 | 0 | 0 | 2.92 |  | 0 | 2.92 | 0 |
|  | 140 | 43 | IE | 5 | 1 | adnexectomy and resection of pelvic LN | 1 | CHOP*6 with etoposide | 0 | 0 | 6.83 |  | 0 | 6.83 | 0 |
|  | 141 | 36 | IE | 1 | 0 |  | 1 | CHOP*6 | 0 | 0 | 6.08 |  | 0 | 6.08 | 0 |
| Demirtas et al. (2020)^67^ | 142 | 46 | IE | 1 | 0 |  | 1 | CHOP*6 | 1 | 0 | 0.5 |  | 0 | 0.5 | 0 |
| Goda et al. (2020)^68^ | 143 | 52 | IE | 1 | 0 |  | 1 | R-CHOP*6 | 1 | 0 | 1.5 |  | 0 | 1.5 | 0 |
|  | 144 | 50 | IE | 1 | 0 |  | 1 | R-CHOP*6 | 1 | 0 | 3.58 |  | 0 | 3.58 | 0 |
|  | 145 | 39 | IE | 1 | 0 |  | 1 | R-CHOP*6 | 1 | 0 | 0.67 |  | 0 | 0.67 | 0 |
|  | 146 | 62 | IIE | 1 | 0 | Para-cervical LN | 1 | R-CEOP*6 | 1 | 0 | 0.83 |  | 0 | 0.83 | 0 |
| Del et a. (2020)^69^ | 147 | 36 | IV | 1 | 0 | Extensive involved cervix, parametrium | 1 | R-CEOP*6 | 0 | 0 | 1.25 |  | 0 | 1.25 | 0 |
| Akkour et al. (2021)^70^ | 148 | 54 | IE | 1 | 1 | radical trachelectomy | 1 | R-CHOP*6 | 1 | 0 | 2 |  | 0 | 2 | 0 |
| Capsa et al. (2022)^71^ | 149 | 75 | IE | 1 | 0 |  | 1 | CHOP*6 | 1 | 0 | 2.42 |  | 0 | 2.42 | 0 |
| Jastaniyah et al. (2013)^72^ | 150 | 54 | IIE | 7 | 0 | Pelvic LN | 0 |  | 1 | 0 | 1.5 |  | 0 | 1.5 | 0 |
| Lovell et a. (2003)^73^ | 151 | 50 | IIE | 7 | 1 | radical hysterectomy  pelvic LN | 0 |  | 1 | 0 | 0.67 |  | 0 | 0.67 | 0 |
| Mansouri et al. (2001) ^74^ | 152 | 34 | IE | 6 | 0 |  | 1 | CHOP*6 | 1 | 0 | 4 |  | 0 | 4 | 0 |
| Singh et al. (2016)^75^ | 153 | 34 | IE | 1 | 0 |  | 1 | R-CHOP*8 | 1 | 0 | 5 |  | 0 | 5 | 0 |
|  | 154 | 77 | IE | 1 | 0 |  | 1 | R-CHOP*6 | 1 | 0 | 3 |  | 0 | 3 | 0 |
| Aozasa et al. (1993) ^76^ | 155 | 30 | IIE | 8 | 1 | Hysterectomy  Pelvic LN | 0 |  | 1 | NA |  |  | 3 | 1.67 | 1 |
|  | 156 | 41 | IIE | 8 | 1 | H/BSO  Pelvic LN | 0 |  | 1 | NA |  |  | 3 | 0.67 | 1 |
|  | 157 | 71 | IE | 8 | 1 | tumor resection | 0 |  | 1 | NA |  |  | 3 | 2 | 0 |
| Bilgin et al. (1999)^77^ | 158 | 74 | IIE | 8 | 0 | Supraclavicular LN | 1 | CHOP*6 | 0 | 0 | 2 |  | 0 | 2 | 0 |
| Venizelos et al. (2003)^78^ | 159 | 34 | IIIEB | 1 | 0 | Lung | 1 | CHOP | 0 | NA |  |  | 1 | 0.83 | 0 |
| Thyagarajan et al. (2004)^79^ | 160 | 41 | IVE | 6 | 0 | Multi organ extensive involved | 1 | unspecified*8 | 1 | 0 | 0.58 |  | 0 | 0.58 | 0 |
| Semczuk et al. (2006) ^80^ | 161 | 43 | IE | 1 | 1 | total hysterectomy | 1 | CHOP*6 | 0 | 0 | 0.83 |  | 0 | 0.83 | 0 |
| Signorelli et al. (2007) ^81^ | 162 | 56 | IIE | 1 | 0 |  | 1 | CHOP*10 | 0 | 0 | 14 |  | 0 | 14 | 0 |
|  | 163 | 29 | IIIE | 1 | 0 |  | 1 | CHOP*6 | 0 | 0 | 10.83 |  | 0 | 10.83 | 0 |
|  | 164 | 32 | IE | 1 | 0 |  | 1 | CHOP*6 | 0 | 0 | 7.58 |  | 0 | 7.58 | 0 |
|  | 165 | 44 | IIE | 1 | 0 |  | 1 | CHOP*6 | 0 | 0 | 7 |  | 0 | 7 | 0 |
|  | 166 | 45 | IE | 1 | 0 |  | 1 | CHOP*6 | 0 | 0 | 3.17 |  | 0 | 3.17 | 0 |
|  | 167 | 46 | IIE | 1 | 0 |  | 1 | CVP*3, CHOP*3 | 0 | 0 | 20.5 |  | 0 | 20.5 | 0 |
|  | 168 | 58 | IE | 1 | 1 | TAH+BSO | 1 | CHOP*6 | 0 | 0 | 19 |  | 0 | 19 | 0 |
|  | 169 | 33 | IIE | 1 | 1 | TAH+BSO | 1 | CHOP*6 | 0 | 0 | 18.92 |  | 0 | 18.92 | 0 |
|  | 170 | 54 | IE | 1 | 1 | polipectomy+H/BSO | 1 | CHOP*6 | 0 | 0 | 9.83 |  | 0 | 9.83 | 0 |
|  | 171 | 69 | IIE | 1 | 1 | TH+BSO+pelvic LN resection+vaginectomy | 1 | CHOP*6 | 1 | 0 | 4.08 |  | 0 | 4.08 | 0 |
| Coon et al. (2008)^82^ | 172 | 56 | IE | 5 | 1 | TAH+BSO | 0 | R*4 | 1 | 0 | 2.33 |  | 0 | 2.33 | 0 |
| Baijal et al. (2009)^83^ | 173 | 44 | IE | 1 | 0 |  | 1 | R-CHOP*3 | 1 | 0 | 1.25 |  | 0 | 1.25 | 0 |
| Novotny et al. (2011)^84^ | 174 | 82 | IE | 1 | 0 |  | 0 |  | 0 | NA |  |  | 3 | 0.08 | 1 |
| Cao et al. (2014)^85^ | 175 | 20 | IE | 1 | 0 |  | 1 | CHOP*7 | 0 | 0 | 7 |  | 0 | 7 | 0 |
|  | 176 | 58 | IE | 1 | 0 |  | 1 | CHOP*6 | 1 | 1 | 3.92 | CNS + systemic relapse | 1 | 4.67 | 0 |
| Igwe et al. (2014)^86^ | 177 | 22 | IIE | 1 | 0 | Pelvic LN | 1 | R-CHOP*5 | 0 | 0 | 0.25 |  | 0 | 0.25 | 0 |

Abbreviations:

OS, overall survival; RFS, recurrent-free survival; DSS, disease-specific survival; R or C, radiotherapy or chemotherapy; Re, recurrence.

CNS, central nervous system; LN, lymph nodes; CDS, cancer-directed surgery.

R-CHOP: rituximab plus cyclophosphamide, doxorubicin, vincristine, and prednisone; COP/CVP, cyclophosphamide, vincristine, and prednisone; R-hyperCVAD, rituximab plus cyclophosphamide, doxorubicin, vincristine, and dexamethasone; BLM, bleomycin; DXM, dexamethasone; MTX, methotrexate; T(A)H+BSO, total (abdominal) hysterectomy plus bilateral salpingo-oophorectomy; R-EPOCH, rituximab plus etoposide, adriamycin, vincristine, cyclophosphamide, and prednisone.

Status: 0, no evidence of disease; 1, alive with disease/partial remission; 2, stable disease/progress disease; 3. Death.

CDS, C, R, Re, DSS: Yes, 1; No, 0; NA, not applicable

Pathological subtypes: 1, diffuse large B-cell lymphoma; 2. Follicular lymphoma; 3. NK/T cell lymphoma; 4. Burkitt lymphoma; 5. Mucosa Associated Lymphoid Tissue (MALT) lymphoma; 6. B-cell lymphoma, unspecified; 7. Hodgkin lymphoma; 8. non-Hodgkin lymphoma, unspecified.

References:

1. Komaki R, Cox JD, Hansen RM, et al. Malignant lymphoma of the uterine cervix. *Cancer* 1984; 54: 1699-1704. 1984/10/15. DOI: 10.1002/1097-0142(19841015)54:8<1699::aid-cncr2820540836>3.0.co;2-e.

2. Bär BM, Reijnders FJ, Keuning JJ, et al. Primary malignant lymphoma of the uterine cervix associated with cold-reacting autoantibody-mediated hemolytic anemia. *Acta Haematol* 1986; 75: 232-235. 1986/01/01. DOI: 10.1159/000206132.

3. Miketic LM, Carroll R, Harris NL, et al. Computed tomography in the evaluation of lymphoma of the uterine cervix. *J Comput Tomogr* 1988; 12: 154-158. 1988/04/01. DOI: 10.1016/0149-936x(88)90073-2.

4. Strang P, Sorbe B and Sundström C. Primary aneuploid lymphoma of the uterine cervix: a case report. *Gynecol Oncol* 1988; 30: 302-305. 1988/06/01. DOI: 10.1016/0090-8258(88)90037-6.

5. Johnston C, Senekjian EK, Ratain MJ, et al. Conservative management of primary cervical lymphoma using combination chemotherapy: a case report. *Gynecol Oncol* 1989; 35: 391-394. 1989/12/01. DOI: 10.1016/0090-8258(89)90086-3.

6. Sandvei R, Lote K, Svendsen E, et al. Successful pregnancy following treatment of primary malignant lymphoma of the uterine cervix. *Gynecol Oncol* 1990; 38: 128-131. 1990/07/01. DOI: 10.1016/0090-8258(90)90024-f.

7. Muntz HG, Ferry JA, Flynn D, et al. Stage IE primary malignant lymphomas of the uterine cervix. *Cancer* 1991; 68: 2023-2032. 1991/11/11. DOI: 10.1002/1097-0142(19911101)68:9<2023::aid-cncr2820680930>3.0.co;2-v.

8. Harris NL and Scully RE. Malignant lymphoma and granulocytic sarcoma of the uterus and vagina. A clinicopathologic analysis of 27 cases. *Cancer* 1984; 53: 2530-2545. 1984/06/01. DOI: 10.1002/1097-0142(19840601)53:11<2530::aid-cncr2820531127>3.0.co;2-j.

9. Broekmans FJ, Swartjes JM, Van der Valk P, et al. Primary malignant lymphoma of the uterus: localization in a cervical polyp. *Eur J Obstet Gynecol Reprod Biol* 1993; 48: 215-219. 1993/03/01. DOI: 10.1016/0028-2243(93)90090-y.

10. Kasales CJ, Langer JE, Young CT, et al. Residual mass after treatment of primary cervical lymphoma: the utility of gallium 67 and MRI. *Abdom Imaging* 1994; 19: 274-277. 1994/05/01. DOI: 10.1007/bf00203527.

11. Awwad JT, Khalil AM, Shamseddine AI, et al. Primary malignant lymphoma of the uterine cervix: is radiotherapy the best therapeutic choice for stage IE? *Gynecol Oncol* 1994; 52: 91-93. 1994/01/01. DOI: 10.1006/gyno.1994.1017.

12. Kuo HC, Chou CY, Chang CH, et al. Primary malignant lymphoma of the uterine cervix shows favorable response to neoadjuvant chemotherapy. *Gynecol Oncol* 1994; 52: 408-410. 1994/03/01. DOI: 10.1006/gyno.1994.1070.

13. Makarewicz R and Kuzminska A. Non-Hodgkin's lymphoma of the uterine cervix: a report of three patients. *Clin Oncol (R Coll Radiol)* 1995; 7: 198-199. 1995/01/01. DOI: 10.1016/s0936-6555(05)80517-5.

14. Patsner B and Greenberg S. Unsuspected primary cervical lymphoma presenting as "cervical fibroid": partial response to leuprolide acetate. *Gynecol Oncol* 1995; 58: 393-394. 1995/09/01. DOI: 10.1006/gyno.1995.1250.

15. Stroh EL, Besa PC, Cox JD, et al. Treatment of patients with lymphomas of the uterus or cervix with combination chemotherapy and radiation therapy. *Cancer* 1995; 75: 2392-2399. 1995/05/01. DOI: 10.1002/1097-0142(19950501)75:9<2392::aid-cncr2820750932>3.0.co;2-y.

16. al-Talib RK, Sworn MJ, Ramsay AD, et al. Primary cervical lymphoma: the role of cervical cytology. *Cytopathology* 1996; 7: 173-177. 1996/06/01. DOI: 10.1046/j.1365-2303.1996.39082390.x.

17. Dhimes P, Alberti N, de Agustín P, et al. Primary malignant lymphoma of the uterine cervix: report of a case with cytologic and immunohistochemical diagnosis. *Cytopathology* 1996; 7: 204-210. 1996/06/01. DOI: 10.1046/j.1365-2303.1996.39182391.x.

18. Chandy L, Kumar L and Dawar R. Non-Hodgkin's lymphoma presenting as a primary lesion in uterine cervix: case report. *J Obstet Gynaecol Res* 1998; 24: 183-187. 1998/08/26. DOI: 10.1111/j.1447-0756.1998.tb00073.x.

19. Clarke DP, Ostler P, Watkinson A, et al. Case report: Magnetic resonance imaging in primary cervical lymphoma: the role in diagnosis and follow-up. *Clin Radiol* 1998; 53: 383-385. 1998/06/18. DOI: 10.1016/s0009-9260(98)80016-0.

20. Lee KM, Seah ES and Sethi VK. Primary non-Hodgkin's lymphoma of the uterine cervix: case report of long-term survival of two patients treated with surgery and radiotherapy. *Australas Radiol* 1998; 42: 126-127. 1998/05/26. DOI: 10.1111/j.1440-1673.1998.tb00587.x.

21. Nasu K, Yoshimatsu J, Urata K, et al. A case of primary non-Hodgkin's lymphoma of the uterine cervix treated by combination chemotherapy (THP-COP). *J Obstet Gynaecol Res* 1998; 24: 157-160. 1998/06/19. DOI: 10.1111/j.1447-0756.1998.tb00068.x.

22. Sungurtekin U, Lacin S and Ayhan S. Primary genital non-Hodgkin lymphoma. *Aust N Z J Obstet Gynaecol* 1998; 38: 346-349. 1998/10/07. DOI: 10.1111/j.1479-828x.1998.tb03086.x.

23. Pelstring RJ, Essell JH, Kurtin PJ, et al. Diversity of organ site involvement among malignant lymphomas of mucosa-associated tissues. *Am J Clin Pathol* 1991; 96: 738-745. 1991/12/01. DOI: 10.1093/ajcp/96.6.738.

24. Rossi G, Bonacorsi G, Longo L, et al. Primary high-grade mucosa-associated lymphoid tissue-type lymphoma of the cervix presenting as a common endocervical polyp. *Arch Pathol Lab Med* 2001; 125: 537-540. 2001/03/22. DOI: 10.5858/2001-125-0537-phgmal.

25. Yokoyama Y, Sato S, Xiao YH, et al. Primary non-Hodgkin's lymphoma of the uterine cervix. *Arch Gynecol Obstet* 2001; 265: 108-111. 2001/06/21. DOI: 10.1007/s004040000147.

26. Au WY, Chan BC, Chung LP, et al. Primary B-cell lymphoma and lymphoma-like lesions of the uterine cervix. *Am J Hematol* 2003; 73: 176-179. 2003/06/27. DOI: 10.1002/ajh.10334.

27. Szánthó A, Bálega JJ, Csapó Z, et al. Primary non-Hodgkin's lymphoma of the uterine cervix successfully treated by neoadjuvant chemotherapy: case report. *Gynecol Oncol* 2003; 89: 171-174. 2003/04/16. DOI: 10.1016/s0090-8258(03)00057-x.

28. Chan JK, Loizzi V, Magistris A, et al. Clinicopathologic features of six cases of primary cervical lymphoma. *Am J Obstet Gynecol* 2005; 193: 866-872. 2005/09/10. DOI: 10.1016/j.ajog.2005.04.044.

29. Perren T, Farrant M, McCarthy K, et al. Lymphomas of the cervix and upper vagina: a report of five cases and a review of the literature. *Gynecol Oncol* 1992; 44: 87-95. 1992/01/01. DOI: 10.1016/0090-8258(92)90018-e.

30. Vang R, Medeiros LJ, Ha CS, et al. Non-Hodgkin's lymphomas involving the uterus: a clinicopathologic analysis of 26 cases. *Mod Pathol* 2000; 13: 19-28. 2000/02/05. DOI: 10.1038/modpathol.3880005.

31. Dursun P, Gultekin M, Bozdag G, et al. Primary cervical lymphoma: report of two cases and review of the literature. *Gynecol Oncol* 2005; 98: 484-489. 2005/07/05. DOI: 10.1016/j.ygyno.2005.04.040.

32. Abbas MA, Birdwell R, Katz DS, et al. Primary lymphoma of the cervix in a heart transplant patient. *AJR Am J Roentgenol* 1996; 167: 1136-1138. 1996/11/01. DOI: 10.2214/ajr.167.5.8911165.

33. Gutman PD, Williams JP, Dveksler GS, et al. T-cell-rich B-cell lymphoma and Epstein-Barr virus infection of the uterus in a postmenopausal patient with an intrauterine contraceptive device in place for over 20 years. *Gynecol Oncol* 1998; 68: 288-292. 1998/05/08. DOI: 10.1006/gyno.1997.4918.

34. Bode MK, Tikkakoski T, Johansson J, et al. Lymphoma of the cervix. Imaging and transcatheter arterial embolization. *Acta Radiol* 2002; 43: 431-432. 2002/09/13. DOI: 10.1080/j.1600-0455.2002.430417.x.

35. Pham DC, Guthrie TH and Ndubisi B. HIV-associated primary cervical non-Hodgkin's lymphoma and two other cases of primary pelvic non-Hodgkin's lymphoma. *Gynecol Oncol* 2003; 90: 204-206. 2003/06/25. DOI: 10.1016/s0090-8258(03)00223-3.

36. Baxter NP, Lane G and Swift S. Primary malignant follicular lymphoma of the cervix: a rare cause of postmenopausal bleeding. *Bjog* 2003; 110: 337-338. 2003/03/12.

37. Kahlifa M, Buckstein R and Perez-Ordoñez B. Sarcomatoid variant of B-cell lymphoma of the uterine cervix. *Int J Gynecol Pathol* 2003; 22: 289-293. 2003/06/24. DOI: 10.1097/01.Pgp.0000070845.25718.4c.

38. Garavaglia E, Taccagni G, Montoli S, et al. Primary stage I-IIE non-Hodgkin's lymphoma of uterine cervix and upper vagina: evidence for a conservative approach in a study on three patients. *Gynecol Oncol* 2005; 97: 214-218. 2005/03/26. DOI: 10.1016/j.ygyno.2004.07.065.

39. Heredia F, Bravo M, Pierotic M, et al. Neoadjuvant combined chemotherapy followed by external whole pelvic irradiation in two cases of primary extranodal non-Hodgkin's lymphoma of the uterine cervix. *Gynecol Oncol* 2005; 97: 285-287. 2005/03/26. DOI: 10.1016/j.ygyno.2004.12.018.

40. Kendrick JEt and Straughn JM, Jr. Two cases of non-Hodgkin's lymphoma presenting as primary gynecologic malignancies. *Gynecol Oncol* 2005; 98: 490-492. 2005/07/05. DOI: 10.1016/j.ygyno.2005.04.033.

41. Frey NV, Svoboda J, Andreadis C, et al. Primary lymphomas of the cervix and uterus: the University of Pennsylvania's experience and a review of the literature. *Leuk Lymphoma* 2006; 47: 1894-1901. 2006/10/27. DOI: 10.1080/10428190600687653.

42. Hariprasad R, Kumar L, Bhatla DM, et al. Primary uterine lymphoma: report of 2 cases and review of literature. *Am J Obstet Gynecol* 2006; 195: 308-313. 2006/07/04. DOI: 10.1016/j.ajog.2006.04.002.

43. Cantú de León D, Pérez Montiel D and Chanona Vilchis J. Primary malignant lymphoma of uterine cervix. *Int J Gynecol Cancer* 2006; 16: 923-927. 2006/05/10. DOI: 10.1111/j.1525-1438.2006.00231.x.

44. Cohn DE, Resnick KE, Eaton LA, et al. Non-Hodgkin's lymphoma mimicking gynecological malignancies of the vagina and cervix: a report of four cases. *Int J Gynecol Cancer* 2007; 17: 274-279. 2007/02/13. DOI: 10.1111/j.1525-1438.2006.00747.x.

45. Korcum AF, Karadogan I, Aksu G, et al. Primary follicular lymphoma of the cervix uteri: a review. *Ann Hematol* 2007; 86: 623-630. 2007/06/23. DOI: 10.1007/s00277-007-0328-0.

46. Lorusso D, Ferrandina G, Pagano L, et al. Successful pregnancy in stage IE primary non-Hodgkin's lymphoma of uterine cervix treated with neoadjuvant chemotherapy and conservative surgery. *Oncology* 2007; 72: 261-264. 2008/01/11. DOI: 10.1159/000113018.

47. Ab Hamid S and Wastie ML. Primary non-Hodgkin's lymphoma presenting as a uterine cervical mass. *Singapore Med J* 2008; 49: e73-75. 2008/03/26.

48. Hanprasertpong J, Hanprasertpong T, Thammavichit T, et al. Primary non-Hodgkin,s lymphoma of the uterine cervix. *Asian Pac J Cancer Prev* 2008; 9: 363-366. 2008/08/21.

49. Upanal N and Enjeti A. Primary lymphoma of the uterus and cervix: two case reports and review of the literature. *Aust N Z J Obstet Gynaecol* 2011; 51: 559-562. 2011/09/20. DOI: 10.1111/j.1479-828X.2011.01365.x.

50. Parva M, Lamb K, Savior DC, et al. Full-term pregnancy and vaginal delivery after treatment for non-Hodgkin's lymphoma of the cervix and lower uterine segment: a case report. *J Obstet Gynaecol Can* 2011; 33: 620-624. 2011/08/19. DOI: 10.1016/s1701-2163(16)34911-8.

51. Ferreri AJ, Verona C, Bolognesi A, et al. Successful pregnancy after chemo-immuno-radiation therapy for aggressive lymphoma of the uterus. *Br J Haematol* 2008; 142: 141-143. 2008/05/15. DOI: 10.1111/j.1365-2141.2008.07083.x.

52. Binesh F, Karimi zarchi M, Vahedian H, et al. Primary malignant lymphoma of the uterine cervix. *BMJ Case Rep* 2012; 2012 2012/09/26. DOI: 10.1136/bcr-2012-006675.

53. Parnis J, Camilleri DJ, Babic D, et al. Lymphoma of the cervix. *Case Rep Hematol* 2012; 2012: 326127. 2012/10/24. DOI: 10.1155/2012/326127.

54. Groszmann Y and Benacerraf BR. Sonographic features of primary lymphoma of the uterine cervix. *J Ultrasound Med* 2013; 32: 717-718. 2013/03/26. DOI: 10.7863/jum.2013.32.4.717.

55. Mandato VD, Palermo R, Falbo A, et al. Primary diffuse large B-cell lymphoma of the uterus: case report and review. *Anticancer Res* 2014; 34: 4377-4390. 2014/07/31.

56. Adachi S, Yamazaki K, Liang SG, et al. Primary uterine diffuse large B-cell lymphoma involving the urinary bladder with urinary cytology mimicking carcinomas: A case report. *J Cytol* 2015; 32: 181-183. 2016/01/06. DOI: 10.4103/0970-9371.168845.

57. Li WS, Wang RC, Wang J, et al. Primary nodular lymphocyte-predominant Hodgkin lymphoma of uterine cervix mimicking leiomyoma. *Clin Case Rep* 2015; 3: 349-352. 2015/07/18. DOI: 10.1002/ccr3.246.

58. Wang GN, Zhao WG, Gao XZ, et al. Primary natural killer/T cell lymphoma of the cervix: case report and clinicopathological analysis. *Taiwan J Obstet Gynecol* 2015; 54: 71-74. 2015/02/14. DOI: 10.1016/j.tjog.2014.11.010.

59. Dobrosavljevic A, Skrobic M, Stanojevic D, et al. Primary non-Hodgkin lymphoma of the uterine cervix of a follicular type - case report. *J Obstet Gynaecol* 2016; 36: 685-686. 2016/04/06. DOI: 10.3109/01443615.2016.1148677.

60. Regalo A, Caseiro L, Pereira E, et al. Primary lymphoma of the uterine cervix: a rare constellation of symptoms. *BMJ Case Rep* 2016; 2016 2016/11/24. DOI: 10.1136/bcr-2016-216597.

61. Zhou W, Hua F, Zuo C, et al. Primary Uterine Cervical Lymphoma Manifesting as Menolipsis Staged and Followed Up by FDG PET/CT. *Clin Nucl Med* 2016; 41: 590-593. 2016/04/08. DOI: 10.1097/rlu.0000000000001196.

62. Cubo AM, Soto ZM, Cruz M, et al. Primary diffuse large B cell lymphoma of the uterine cervix successfully treated by combined chemotherapy alone: A case report. *Medicine (Baltimore)* 2017; 96: e6846. 2017/05/11. DOI: 10.1097/md.0000000000006846.

63. Kosari F, Niknejad N, Nili F, et al. Peripheral T-Cell Lymphoma Presenting as a Primary Uterine Cervix Mass: A Report of a Rare Case. *Int J Gynecol Pathol* 2017; 36: 523-527. 2017/03/01. DOI: 10.1097/pgp.0000000000000358.

64. Omori M, Oishi N, Nakazawa T, et al. Extranodal NK/T-cell lymphoma, nasal type of the uterine cervix: A case report. *Diagn Cytopathol* 2016; 44: 430-433. 2016/02/13. DOI: 10.1002/dc.23439.

65. Roberts ME and Cottrill HM. A case of primary cervical lymphoma in a patient with abnormal uterine bleeding. *Gynecol Oncol Rep* 2018; 26: 105-107. 2018/12/12. DOI: 10.1016/j.gore.2018.10.014.

66. Gui W, Li J, Zhang Z, et al. Primary hematological malignancy of the uterine cervix: A case report. *Oncol Lett* 2019; 18: 3337-3341. 2019/09/14. DOI: 10.3892/ol.2019.10652.

67. Selvi Demirtas G, Gokcu M, Sancı M, et al. Primary non-Hodgkin's lymphoma masquerading as cervical cancer. *Ginekol Pol* 2020; 91: 571. 2020/10/09. DOI: 10.5603/gp.2020.0090.

68. Goda JS, Gaikwad U, Narayan A, et al. Primary diffuse large B cell lymphoma of Uterine Cervix: Treatment outcomes of a rare entity with literature review. *Cancer Rep (Hoboken)* 2020; 3: e1264. 2020/08/08. DOI: 10.1002/cnr2.1264.

69. Del M, Angeles MA, Syrykh C, et al. Primary B-Cell lymphoma of the uterine cervix presenting with right ureter hydronephrosis: A case report. *Gynecol Oncol Rep* 2020; 34: 100639. 2020/10/01. DOI: 10.1016/j.gore.2020.100639.

70. Akkour K, Alhulwah M, Alhalal H, et al. Primary extranodal diffuse large B-cell lymphoma of the uterine cervix. *Malays J Pathol* 2021; 43: 327-331. 2021/08/28.

71. Capsa C, Calustian LA, Antoniu SA, et al. Primary Non-Hodgkin Uterine Lymphoma of the Cervix: A Literature Review. *Medicina (Kaunas)* 2022; 58 2022/01/22. DOI: 10.3390/medicina58010106.

72. Jastaniyah N, Lai R and Pearcey R. Nodular lymphocyte predominant Hodgkin's lymphoma of the cervix: A case report of a rare entity. *Gynecol Oncol Case Rep* 2012; 4: 4-6. 2012/01/01. DOI: 10.1016/j.gynor.2012.11.001.

73. Lovell MO and Valente PT. Unique collision of hodgkin lymphoma and adenosquamous carcinoma in the uterine cervix: synchronous malignant neoplasms of the cervix. *J Low Genit Tract Dis* 2003; 7: 307-310. 2006/10/20. DOI: 10.1097/00128360-200310000-00015.

74. Mansouri H, Kebdani T, Hassouni K, et al. Unusual locations for lymphomas. Case 1. Intermediate-grade lymphoma of the cervix. *J Clin Oncol* 2001; 19: 2959-2960. 2001/06/02. DOI: 10.1200/jco.2001.19.11.2959.

75. Singh L, Madan R, Benson R, et al. Primary Non-Hodgkins Lymphoma of Uterine Cervix: A Case Report of Two Patients. *J Obstet Gynaecol India* 2016; 66: 125-127. 2016/04/06. DOI: 10.1007/s13224-014-0647-8.

76. Aozasa K, Saeki K, Ohsawa M, et al. Malignant lymphoma of the uterus. Report of seven cases with immunohistochemical study. *Cancer* 1993; 72: 1959-1964. 1993/09/15. DOI: 10.1002/1097-0142(19930915)72:6<1959::aid-cncr2820720628>3.0.co;2-p.

77. Bilgin T, Doş A and Tolunay S. Primary malignant lymphoma of the uterine cervix: difficulties in diagnosis. *J Obstet Gynaecol* 1999; 19: 671-672. 2004/10/30. DOI: 10.1080/01443619964085.

78. Venizelos ID, Zafrakas M, Dragoumis K, et al. Non-Hodgkin's lymphoma involving the uterine cervix after treatment for Hodgkin disease. *Leuk Lymphoma* 2003; 44: 2155-2157. 2004/02/13. DOI: 10.1080/1042819031000116643.

79. Thyagarajan MS, Dobson MJ and Biswas A. Case report: appearance of uterine cervical lymphoma on MRI: a case report and review of the literature. *Br J Radiol* 2004; 77: 512-515. 2004/05/21. DOI: 10.1259/bjr/58044417.

80. Anagnostopoulos A, Mouzakiti N, Ruthven S, et al. Primary cervical and uterine corpus lymphoma; a case report and literature review. *Int J Clin Exp Med* 2013; 6: 298-306. 2013/05/04.

81. Signorelli M, Maneo A, Cammarota S, et al. Conservative management in primary genital lymphomas: the role of chemotherapy. *Gynecol Oncol* 2007; 104: 416-421. 2006/10/20. DOI: 10.1016/j.ygyno.2006.08.024.

82. Coon D, Beriwal S, Swerdlow SH, et al. Mucosa-associated lymphoid tissue lymphoma of the cervix. *J Clin Oncol* 2008; 26: 503-504. 2008/01/19. DOI: 10.1200/jco.2007.14.3784.

83. Baijal G, Vadiraja BM, Fernandes DJ, et al. Diffuse large B-cell lymphoma of the uterine cervix: a rare case managed novelly. *J Cancer Res Ther* 2009; 5: 140-142. 2009/06/23. DOI: 10.4103/0973-1482.52784.

84. Novotny S, Ellis T and Stephens J. Primary B-cell lymphoma of the cervix presenting with bilateral hydronephrosis. *Obstet Gynecol* 2011; 117: 444-446. 2011/01/22. DOI: 10.1097/AOG.0b013e3181f1f2ec.

85. Cao XX, Li J, Zhang W, et al. Patients with primary diffuse large B-cell lymphoma of female genital tract have high risk of central nervous system relapse. *Ann Hematol* 2014; 93: 1001-1005. 2014/01/11. DOI: 10.1007/s00277-013-2003-y.

86. Igwe E, Diaz J and Ferriss J. Diffuse large B cell lymphoma of the cervix with rectal involvement. *Gynecol Oncol Rep* 2014; 10: 1-4. 2015/06/16. DOI: 10.1016/j.gore.2014.07.004.
