# Supplementary material for: Advanced Ann Arbor stage and age over 60 years as prognostic predictors in patients with primary cervical lymphoma: a retrospective cohort study and systematic review
Source: BMC Cancer. 2023 Jan 27;23:95. doi: 10.1186/s12885-023-10548-4 (PMC9881271; doi:10.1186/s12885-023-10548-4)
Supplement: Supplementary file 3 — Additional file 3: Table S2a. Univariate and multivariate analysis of OS in DLBCL population. Table S2b. Univariate and multivariate analysis of DSS in DLBCL population. Table S2c. Univariate and multivariate analysis of RFS in DLBCL population (N = 101). [file 12885_2023_10548_MOESM3_ESM.docx]

**Table S2a** Univariate and multivariate analysis of OS in DLBCL population.

| Factors | N |  | Univariate analysis | |  | Multivariate cox regression analysis | | | |
| --- | --- | --- | --- | --- | --- | --- | --- | --- | --- |
|  |  | Mean survival(y) | 5-year survival rate | p |  | OR | (95% CI) | | p |
| Age (<60/≥60, years) ^a, *^ | 82/31 | 19.4/8.4 | 96.1%/63.6% | <0.001 |  | 26.324 | | 5.090-136.144 | <0.001 |
| Ann Arbor stage (I-IIE/III-IVE) ^a, *^ | 98/15 | 18.3/5.6 | 91.7%/49.7% | <0.001 |  | 4.219 1.314-13.551 0.016 | | | |
| Cancer-directed surgery (No/Yes) | 82/31 | 16.8/16.6 | 84.6%/92.8% | 0.483 |  |  | | | |
| Chemotherapy/radiotherapy ^a, *^ |  |  |  | 0.006 |  | 0.008 | | | |
| No/R or C | 6/70 | 5.0/16.7 | 66.7%/83.5% | 0.027 |  | 0.081 0.013-0.509 0.007 | | | |
| No/R + C | 6/37 | 5.0/14.0 | 66.7%/97.1% | <0.001 |  | 0.018 0.001-0.267 0.003 | | | |
| R or C / R + C | 70/37 | 16.7/14.0 | 83.5%/97.1% | 0.094 |  | 0.227 0.028-1.810 0.161 | | | |

a, Factors applied to multivariate analysis; *, p < 0.05

**Table S2b** Univariate and multivariate analysis of DSS in DLBCL population.

| Factors | N |  | Univariate analysis | |  | Multivariate cox regression analysis | | | |
| --- | --- | --- | --- | --- | --- | --- | --- | --- | --- |
|  |  | Mean survival(y) | 5-year DSS rate | p |  | OR | (95% CI) | | p |
| Age (<60/≥60, years) ^a, *^ | 82/31 | 19.8/9.5 | 96.1%/65.9% | <0.001 |  | 23.015 | | 3.857-137.324 | 0.001 |
| Ann Arbor stage (I-IIE/III-IVE) ^a, *^ | 98/15 | 19.1/6.4 | 92.7%/49.7% | <0.001 |  | 4.056 1.137-14.469 0.031 | | | |
| Cancer-directed surgery (No/Yes) | 82/31 | 17.8/17.7 | 85.7%/92.8% | 0.403 |  |  | | | |
| Chemotherapy/radiotherapy ^a, *^ |  |  |  | 0.056 |  | 0.049 | | | |
| No/R or C | 6/70 | 6.0/17.7 | 66.7%/84.8% | 0.131 |  | 0.120 0.016-0.893 0.038 | | | |
| No/R + C | 6/37 | 6.0/14.0 | 66.7%/97.1% | 0.004 |  | 0.034 0.002-0.562 0.018 | | | |
| R or C / R + C | 70/37 | 17.7/14.0 | 84.8%/97.1% | 0.160 |  | 0.282 0.034-2.344 0.241 | | | |

a, Factors applied to multivariate analysis; *, p < 0.05.

**Table S2c** Univariate and multivariate analysis of RFS in DLBCL population (N = 101).

| Factors | N |  | Univariate analysis | |  | Multivariate cox regression analysis | | | |
| --- | --- | --- | --- | --- | --- | --- | --- | --- | --- |
|  |  | Mean RFS (y) | 5-year RFS rate | p |  | OR | (95% CI) | | p |
| Age (<60/≥60, years) | 78/23 | 18.2/11.4 | 87.6%/82.4% | 0.287 |  |  | |  |  |
| Ann Arbor stage (I-IIE/III-IVE) ^a, *^ | 90/11 | 18.4/7.2 | 88.9%/61.4% | 0.031 |  |  | | | |
| Cancer-directed surgery (No/Yes) | 72/29 | 18.0/16.4 | 86.7%/86.1% | 0.511 |  |  | | | |
| Chemotherapy/radiotherapy ^a, *^ |  |  |  | <0.001 |  | 0.003 | | | |
| No/R or C | 3/65 | 3.1/17.7 | 33.3%/85.2% | <0.001 |  | 0.100 0.021-0.480 0.004 | | | |
| No/R + C | 3/33 | 3.1/13.8 | 33.3%/93.8% | <0.001 |  | 0.025 0.002-0.275 0.003 | | | |
| R or C / R + C | 65/33 | 17.7/13.8 | 85.2%/93.8% | 0.150 |  | 0.246 0.031-1.963 0.186 | | | |

a, Factors applied to multivariate analysis; *, p < 0.05.

Abbreviations: DLBCL, diffuse large B-cell lymphoma; OS, overall survival; RFS, recurrent-free survival; DSS, disease-specific survival; R or/+ C, radiotherapy or/plus chemotherapy.
