# Supplementary material for: Advanced Ann Arbor stage and age over 60 years as prognostic predictors in patients with primary cervical lymphoma: a retrospective cohort study and systematic review
Source: BMC Cancer. 2023 Jan 27;23:95. doi: 10.1186/s12885-023-10548-4 (PMC9881271; doi:10.1186/s12885-023-10548-4)
Supplement: Supplementary file 5 — Additional file 5: Table S3a. Univariate and multivariate analysis of OS in DLBCL population (Ann Arbor stage IE). Table S3b. Univariate and multivariate analysis of DSS in DLBCL population (Ann Arbor stage IE). Table S3c. Univariate and multivariate analysis of RFS in DLBCL population (Ann Arbor stage IE). [file 12885_2023_10548_MOESM5_ESM.docx]

**Table S3a** Univariate and multivariate analysis of OS in DLBCL population (Ann Arbor stage IE).

| Factors | N | Univariate analysis | |  | Multivariate cox regression analysis | | | |
| --- | --- | --- | --- | --- | --- | --- | --- | --- |
|  |  | 5-year survival rate | p |  | OR | (95% CI) | | p |
| Age (<60/≥60, years) ^a, *^ | 50/23 | 100%/73.0% | 0.001 |  |  | |  | 0.147 |
| CDS (No/Yes) | 53/20 | 91.7%/89.5% | 0.809 |  |  | | | |
| Chemotherapy/radiotherapy ^a^ |  |  | 0.165 |  |  | | | |
| No/R or C | 4/45 | 75.0%/87.7% | 0.518 |  |  | | | |
| No/R + C | 4/24 | 75.0%/100% | 0.014 |  |  | | | |
| R or C / R + C | 45/24 | 87.7%/100% | 0.098 |  |  | | | |

a, Factors applied to multivariate analysis; *, p < 0.05

**Table S3b** Univariate and multivariate analysis of DSS in DLBCL population (Ann Arbor stage IE).

| Factors | N | Univariate analysis | |  | Multivariate cox regression analysis | | | |
| --- | --- | --- | --- | --- | --- | --- | --- | --- |
|  |  | 5-year DSS rate | p |  | OR | (95% CI) | | p |
| Age (<60/≥60, years) ^a, *^ | 50/23 | 100%/76.5% | 0.001 |  |  | |  | 0.221 |
| CDS (No/Yes) | 53/20 | 93.5%/89.5% | 0.637 |  |  | | | |
| Chemotherapy/radiotherapy ^a^ |  |  | 0.140 |  |  | | | |
| No/R or C | 4/45 | 75.0%/89.7% | 0.294 |  |  | | | |
| No/R + C | 4/24 | 75.0%/100% | 0.014 |  |  | | | |
| R or C / R + C | 45/24 | 89.7%/100% | 0.160 |  |  | | | |

a, Factors applied to multivariate analysis; *, p < 0.05.

**Table S3c** Univariate and multivariate analysis of RFS in DLBCL population (Ann Arbor stage IE).

| Factors | N | Univariate analysis | |  | Multivariate cox regression analysis | | |
| --- | --- | --- | --- | --- | --- | --- | --- |
|  |  | 5-year RFS rate | p |  | OR | (95% CI) | p |
| Age (<60/≥60, years) ^a^ | 47/18 | 93.2%/83.3% | 0.113 |  |  |  |  |
| CDS (No/Yes) | 47/18 | 90.8%/88.9% | 0.520 |  |  | | |
| Chemotherapy/radiotherapy ^a, *^ |  |  | 0.029 |  | 0.105 | | |
| No/R or C | 2/42 | 50.0%/87.5% | 0.009 |  |  | | |
| No/R + C | 2/21 | 50.0%/92.6% | 0.057 |  |  | | |
| R or C / R + C | 42/21 | 87.5%/92.6% | 0.768 |  |  | | |

a, Factors applied to multivariate analysis; *, p < 0.05.

Abbreviations: DLBCL, diffuse large B-cell lymphoma; CDS, Cancer-directed surgery; OS, overall survival; RFS, recurrent-free survival; DSS, disease-specific survival; R or/+ C, radiotherapy or/plus chemotherapy.
